# Cryptic sulfur cycling in the deep biosphere of ferruginous Lake Towuti, Indonesia

**DOI:** 10.3389/fmicb.2025.1725877

**Published:** 2025-12-11

**Authors:** Fátima Ruiz-Blas, Alexander Bartholomäus, Cynthia Henny, James M. Russell, Jens Kallmeyer, Aurèle Vuillemin

**Affiliations:** 1 GFZ Helmholtz Centre for Geosciences, Section Geomicrobiology, Potsdam, Germany; 2 Research Center for Limnology and Water Resources, National Research and Innovation Agency (BRIN), Cibinong, Jawa Barat, Indonesia; 3 Department of Earth, Environmental, and Planetary Sciences, Brown University, Providence, RI, United States; 4 Faculty of Biology, University Duisburg-Essen, Essen, Germany

**Keywords:** ferruginous conditions, cryptic sulfur cycle, metagenomes, reactive iron phases, pore water geochemistry, Bathyarchaeia, Lake Towuti, International Continental scientific Drilling Program (ICDP)

## Abstract

Lake Towuti, Sulawesi, Indonesia is an ancient tectonic lake, exhibiting iron-rich, sulfate-poor anoxic deep waters. Temporal variations in water column stratification led to sediment accumulation under variable redox conditions. Such ferruginous settings make Lake Towuti an ideal study site to evaluate how a cryptic sulfur cycle could possibly operate under a scarcity of sulfate and abundance of iron minerals, similar to Earth’s primitive oceans. Here, we integrate downcore profiles for pore water geochemistry, reactive iron mineralogy, and bulk sediment elemental composition with microbial cell counts, sulfate reduction rates, 16S rRNA genes and metagenomes to resolve microbial sulfur transformations down to 15 m below lake floor (mblf). Sulfate concentrations and reduction rates dropped within the upper mblf, while pore water ferrous iron increased to its highest concentration down to 3 mblf. Any microbially-produced sulfide precipitated as reduced inorganic sulfur in the sediment, apparently forming authigenic millerite (NiS) during burial. The decrease in cell densities tracked the decline in electron acceptors in pore waters with depth. From 3 to 10 mblf, low but sustained sulfate reduction rates were observed with intermittent presence of nitrate in pore water and increased goethite in the sediment, both acting as potential oxidants of sulfur intermediates. A subsequent re-increase in pore water sulfate occurred in parallel with syntrophic fermentation of volatile fatty acids. Consistent with geochemical evolution, the taxonomic diversity of microbial populations shifted from a bacterial assemblage near the surface to selective but prevailing Bathyarchaeia down to 15 mblf. The corresponding metagenome-assembled genomes predicted metabolic potential for complete sulfate reduction (*aprAB*, *dsrAB*) in Thermodesulfovibrionia, whereas Desulfobacterota (incl. Geobacterales, Desulfuromonadales, Syntrophales) and Aminicenantia exhibited versatility in reducing iron, nitrate (*narG*, *napA*), nitrite (*nirS*, *nrfA*) and sulfate (*dsrAB*, *asrA*). By contrast, Bathyarchaeia were predicted to disproportionate sulfur to polysulfides and reduce ferredoxin via electron bifurcation (*hyd I-II*, *sudA*, *dsrC*, *dsrE*) to fuel a Wood-Ljungdahl pathway, defining homoacetogenesis as terminal electron sink. Together, these mineralogical, geochemical, and metagenomic features provide evidence for a spatially confined but active cryptic sulfur cycle with tight coupling between reduction of mineral ferric iron and intermittent pore water nitrate to syntrophic and lithotrophic (homo)acetogenesis.

## 1 Introduction

**Figure 1:**
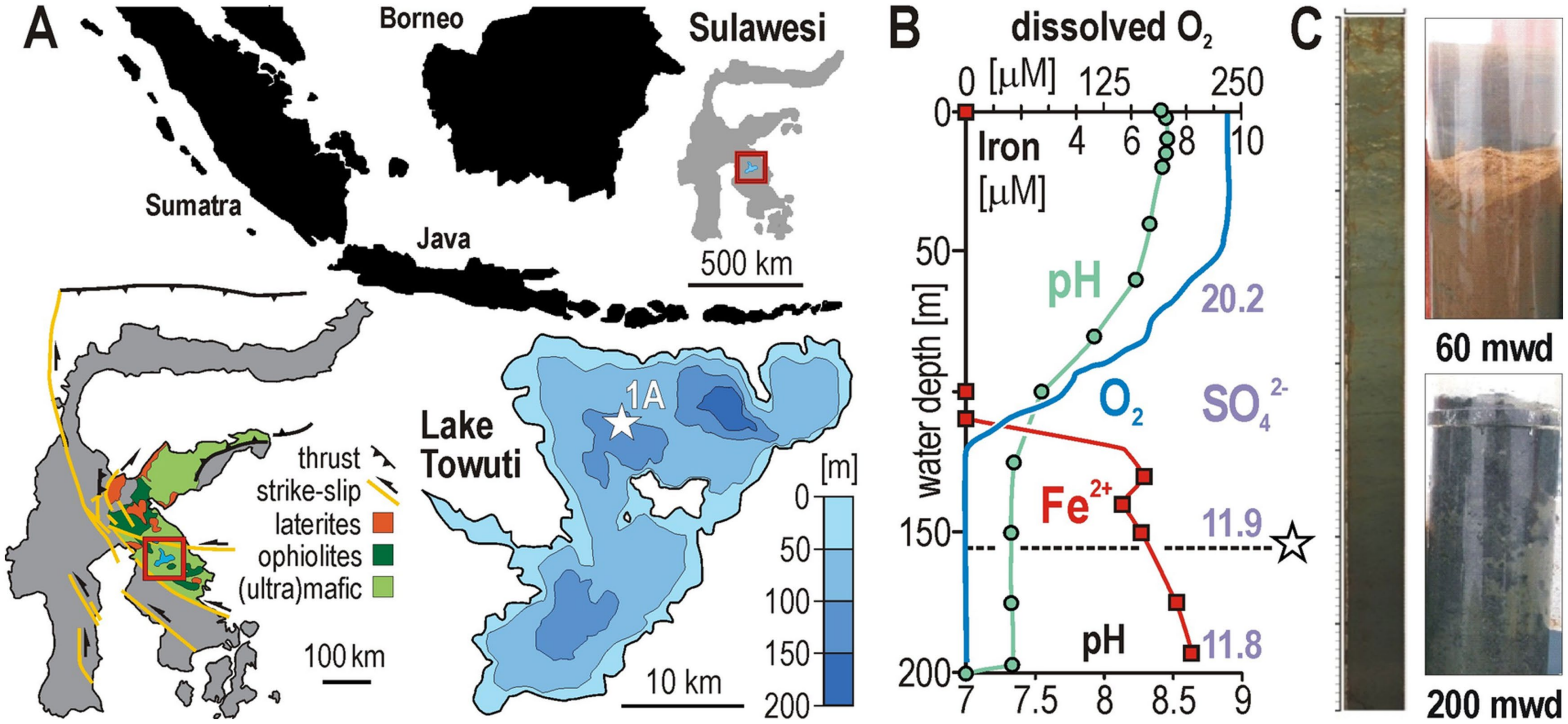
Site description and water-column chemistry of Lake Towuti. **(A)** View on the Indonesian archipelago with Sulawesi Island and location of Lake Towuti (square); geological map of the catchment with the Malili Lake system (square); and bathymetry of Lake Towuti with location of the drilling site (star). **(B)** Water column vertical profiles for pH (dots), dissolved O_2_ (line), Fe^2+^ (squares), and SO_4_^2−^ (numbers) concentrations. The dashed line right below the oxycline marks the depth at which the hydraulic core TDP-1A (star) was retrieved (i.e., 156 m water depth). **(C)** Representative sediment core section and gravity cores retrieved from 60 m and 200 m water depth (mwd), illustrating how redox conditions at the sediment–water interface result in an alternation of red (i.e., oxic) and green (i.e., anoxic) clay. Adapted from Vuillemin et al. (2023b), licensed under CC BY 4.0.

Ferruginous conditions, defined by anoxia with high concentrations of dissolved Fe^2+^ and limited availability of SO_4_^2−^ (Crowe et al., 2014), were widespread in Precambrian oceans and largely influenced the evolution of microbial redox processes over geological times (Poulton and Canfield, 2011; Lyons et al., 2014). While ferruginous conditions are restricted today, they persist in isolated freshwater ecosystems (Koeksoy et al., 2016), offering a time window into ancient biogeochemical cycles (Berg et al., 2019; Swanner et al., 2020). Modern Lake Towuti (Sulawesi, Indonesia) constitutes a suitable ferruginous analogue in which to investigate microbial processes under dynamic redox conditions and during long-term confinement in the subsurface (Ruiz-Blas et al., 2025). Lateritic erosion of ultramafic rocks in the catchment (Figure 1A) delivers abundant reactive iron oxides, with a scarcity of dissolved SO_4_^2−^ (typically <20 μM). The 203-m-deep basin is stratified, with a water column that remains anoxic below 130 m (Figure 1B), although sporadic mixing has been reported (Costa et al., 2015; Pu et al., 2025). Thus, redox conditions at the sediment–water interface likely fluctuated (Figure 1C) as sediments accumulated over Quaternary cycles (Russell et al., 2020).

Due to limited availability of phosphate, which restricts productivity (Zegeye et al., 2012), and respiratory electron acceptors in the water column, ferruginous settings and their iron-rich deposits represent a low-energy sedimentary environment for microbes. As fresh sediments accumulate, canonical respiratory types proceed according to a decreasing energy yield (i.e., O_2_, NO_3_^−^, Mn^4+^, Fe^3+^, SO_4_^2−^, CO_3_^−^) and pore water availability of terminal electron acceptors, ending with CO_2_ fixation via methanogenesis and/or homoacetogenesis (Esteban et al., 2015). In the absence of external oxidants, organic matter is degraded stepwise via syntrophic fermentation, during which the production and consumption of volatile fatty acids ultimately releases acetate, H_2_, CO_2_ and methane in the pore water (Sieber et al., 2012). Such syntrophic interactions proceed through a layering of microbial populations with sediment depth and result in an accumulation of dissolved inorganic carbon (DIC) and methane as end products (McInerney et al., 2009). This results in a steep downcore decline in cell abundances (Kallmeyer et al., 2012, 2015) and reduced population turnover among the subsurface biosphere due to serious substrate depletion (Jørgensen and Marshall, 2016).

By definition, ferruginous conditions imply a steep redox gradient in sediments inherent to limited pore water availability of specific electron acceptors (i.e., O_2_, NO_3_^−^, SO_4_^2−^) in sediments. Moreover, microbial use of structural Fe(III) in iron oxides-hydroxides as electron acceptors is highly dependent on their reactivity towards dissimilatory iron reduction (Bauer et al., 2020; Friese et al., 2021). Instead, it was suggested that, under ferruginous conditions, mineral ferric iron could drive the reoxidation of intermediate sulfur species (i.e., SO_3_^2−^, S_2_O_3_^2−^, S_2_O_6_^2−^, S_X_^0^, HS^−^) and their consumption as both electron acceptors and donors (Vuillemin et al., 2018; Berg et al., 2019). This unique use of sulfur as electron donor and acceptor (i.e., disproportionation) has been termed “inorganic fermentation” (Jørgensen, 2021), and the related cryptic reactions are known to proceed at appreciable rates despite low sulfate concentrations (Zopfi et al., 2004; Jørgensen et al., 2019), potentially sustaining lithotrophy in the deep subsurface. Alternatively, re-oxidation of reduced and organic sulfur as an electron donor can also be coupled to the dissimilatory reduction of ferric iron and nitrate/nitrite (Berg et al., 2025; Patsis et al., 2025), involving a network of interdependent redox reactions shaped by electron donor and acceptor availability (Jørgensen et al., 2019). However, cryptic sulfur cycling involves rapid turnover of reduced sulfur species (like sulfide) through reoxidation pathways (Zopfi et al., 2004; Hansel et al., 2015), with little pore water geochemical signal (Berg et al., 2019; Jørgensen et al., 2019), but a faint mineralogical imprint in the form of iron sulfides, generally pyrite (FeS_2_).

Recent amplicon surveys and metagenomes have begun to document Lake Towuti’s shallow subsurface biosphere. Surface sediments are densely populated by Chloroflexota, Acidobacteriota and Desulfobacterota (Vuillemin et al., 2024), whereas archaeal lineages, especially Bathyarchaeia (Ruiz-Blas et al., 2024), clearly prevail below the sulfate-reduction zone about 0.5 m below lake floor (mblf) and deeper into the fermentative zone (Ruiz-Blas et al., 2025). Metagenome-assembled genome (MAGs) analyses could assign functional guilds of iron reducers (FeRB) and sulfate reducers (SRB) to Aminicenantia, Desulfobacterota, and Nitrospirota, respectively, yet only fragmentary evidence exists for sulfur-disproportionation pathways across these communities (Vuillemin et al., 2018). Below 0.5 mblf, Bathyarchaeia evolve as mixotrophic homoacetogens encoding a complete Wood-Ljungdahl pathway (WLP) with specific ferredoxin hydrogenases that would enable them to harness redox energy from molecular hydrogen and sulfur (Ruiz-Blas et al., 2024).

Here, we specifically address the presence of a cryptic sulfur cycle in the ferruginous subsurface, producing novel metagenome-resolved analyses in their direct geochemical and mineralogical context, documenting sulfate reduction rates (SRR), total reduced inorganic sulfur, pore water geochemistry, speciation of reactive iron phases, and X-ray fluorescence core scanning profiles along Lake Towuti’s upper 15 mblf of sediment. This sediment sequence, cored by the International Continental scientific Drilling Program (ICDP), recorded some 80 ka of environmental history (Ulfers et al., 2021). In combination with mineralogical (Morlock et al., 2021; Sheppard et al., 2021) and geochemical data (Friese et al., 2021; Vuillemin et al., 2023a), we explore how key functional genes, mainly those associated with dissimilatory iron and sulfate reduction, sulfur cycling, and redox transformations of fixed nitrogen, are distributed among microbial populations in the deep ferruginous subsurface. We aim to identify which metabolic pathways can drive a cryptic sulfur cycle under substrate-limited ferruginous anoxia and efficiently harness redox energy to fuel putative lithoautotrophs in the deep biosphere.

## 2 Materials and methods

### 2.1 Site description and sampling operations

Lake Towuti (2°45′S, 121°30′E), located in Central Sulawesi, Indonesia, is the largest of the Malili Lakes. With a surface area of ca. 561 km^2^ and a maximum depth of 203 meters (Figure 1A), it is one of the largest and deepest tectonic lakes in Southeast Asia. The lake sedimentary archive recorded late Quaternary fluctuations in lake level, productivity, and redox state (Figure 1C) linked to regional climate and tectonics, as shown by drilling-based stratigraphy and downcore proxies (Russell et al., 2020; Morlock et al., 2021; Sheppard et al., 2021; Vuillemin et al., 2023a).

The water column dissolved oxygen profile was acquired with an SBE-19 Conductivity-Temperature-Depth profiler (Sea-Bird Scientific, Bellevue, United States). Water samples from discrete depths were collected using 5-L Niskin bottles (General Oceanics, Miami, United States) deployed and positioned using a FCV-585 echosounder (Furuno Electric, Nishinomiya, Japan). Immediately upon recovery, pH was measured with a WTW 3310 handheld meter (Thermo Fisher Scientific, Waltham, United States), and dissolved Fe^2+^ was quantified on a DR 3900 spectrophotometer (Hach, Düsseldorf, Germany) using the ferrozine method (Viollier et al., 2000; Bauer et al., 2020). Surface sediments were obtained via gravity coring in November 2013 and 2014, and processed on site in an anoxic glove bag for pore water extraction and DNA sampling (Vuillemin et al., 2016).

In May to July 2015, the core TDP-1A was retrieved via hydraulic piston coring at 156 m water depth using the ICDP’s Deep Lake Drilling System (Russell et al., 2016). A contamination tracer was added to the drilling fluid to facilitate sampling of pristine core sections for geomicrobiology (Friese et al., 2017). Core sections with visible signs of the tracer were excluded from downstream analyses. After retrieval, whole-round cores were cut with the liners, capped, transferred into a nitrogen-flushed glove box, and subsampled for pore water extraction, total cell counts, sulfate reduction rates, and DNA extraction, as previously detailed (Russell et al., 2016; Vuillemin et al., 2023a). Samples for genomic analyses were taken by placing whole-round cores in aluminum foil bags, flushed with N_2_ gas, and heat-sealed. In January 2016, the remainders of core sections were split, scanned, and subsampled for mineralogical and sedimentological work (Russell et al., 2016, 2020; Vuillemin et al., 2019, 2020, 2023a,b) at the National Lacustrine Core Facility (LacCore), University of Minnesota.

### 2.2 X-ray fluorescence core scanning elemental analysis

Elemental profiles (i.e., Ti, S, Ni, Fe, Mn) were measured on split core sections, using an ITRAX X-ray fluorescence core scanner (Cox Analytical Systems, Mölndal, Sweden). Scanning was conducted at 5 mm resolution with Cr and Mo X-ray tubes (30 kV, 50 mA, 50 s integration time) to enhance the detection of low and high atomic weight elements, respectively. Data were post-processed using a multivariate log-ratio calibration (MCL) algorithm to estimate elemental concentrations in weight percent (Morlock et al., 2021).

### 2.3 Sulfate reduction rates, total reduced sulfur, and reactive iron phases

Potential sulfate reduction rates were determined following the established protocol (Kallmeyer et al., 2004). Sediment samples were incubated with radioactive ^35^SO_4_^2−^ in glass barrels fitted with a syringe plunger on one end and a butyl rubber stopper on the other. Samples were taken in triplicates. Microbially reduced inorganic sulfur species were extracted using cold chromium distillation (Kallmeyer et al., 2004). Radioactivity was quantified via liquid scintillation counting using Ultima Gold Scintillation Cocktail and a Tri-Carb 2,500 TR counter (both Perkin Elmer, Shelton, United States).

Iron speciation was analyzed following the established protocol (Poulton and Canfield, 2005). Wet sediment subsamples (500 mg) were extracted immediately in the field with 1 mL of 0.5 N HCl, and Fe(II) and Fe(III) in the easily extractable pool were measured on site via a ferrozine spectrophotometric assay (Viollier et al., 2000). For sequential extraction of reactive and total Fe, anoxically preserved, freeze-dried sediments were ground to fine powder and processed in 200 mg aliquots. The highly reactive Fe pool includes hydrous Fe (oxyhydr)oxides (ferrihydrite, lepidocrocite), carbonate-associated Fe (siderite), ferric (oxyhydr)oxides (hematite, goethite), and magnetite extracted sequentially with 0.5 N HCl, sodium acetate, sodium dithionite, and ammonium oxalate, respectively. Non-reactive, mostly silicate-bound Fe was extracted using near-boiling 6 N HCl (Bauer et al., 2020). Pyrite is excluded from these fractions (Henkel et al., 2018).

### 2.4 Pore water geochemistry

Pore water concentrations of Cl^−^, SO₄^2−^, NO_3_^−^, Na^+^, Mg^2+^, Ca^2+^, and NH₄^+^ were measured using an ion chromatograph (Sykam, Fürstenfeldbruck, Germany) equipped with SeQuant SAMS suppressor (EMD Millipore, Billerica, United States) for anions and non-suppressed IC system for cations, as published (Vuillemin et al., 2016, 2020). Concentrations of PO₄^3−^, Fe^2+^, and Mn^2+^ were determined via spectrophotometry (Hach) following established protocols (Vuillemin et al., 2023a). Trace metals (i.e., As, Co) were measured on pore water samples acidified with 2% HNO_3_ using high-resolution inductively coupled plasma mass spectrometry (HR-ICP-MS; Thermo Fisher Scientific), and quantified against internal standards (Vuillemin et al., 2023a).

Pore water concentrations in volatile fatty acids (i.e., lactate, formate, acetate, propionate, butyrate) were measured using two-dimensional ion chromatography coupled to mass spectrometry (2D IC-MS). Analyses were performed on a Dionex ICS-3000 ion chromatograph coupled to a Surveyor MSQ Plus mass spectrometer (both Thermo Fisher Scientific) and quantified against external standards, as previously published (Glombitza et al., 2014).

For methane concentrations, 3 cm^3^ of sediments were collected immediately after core retrieval using end-cut syringes, transferred to vials prefilled with a saturated NaCl solution, and sealed without headspace. In the lab, we created a small He headspace, equilibrated for ca. 24 h, and methane concentrations were quantified on a Thermo Finnigan trace gas chromatograph equipped with a flame ionization detector (Thermo Fisher Scientific), as published (Heuer et al., 2009).

### 2.5 Microbial cell counts, and DNA extractions

Total cell counts were determined on 2 cm^3^ of sediment fixed in formalin (2% final concentration). Fifty mL of sediment slurry were mixed with 50 mL of detergent solution (i.e., 36.8 g L^−1^ Na_2_ EDTA × 2 H_2_O, 22.3 g L^−1^ Na-pyrophosphate × 10 H_2_O, 5 mL TWEEN 80), processed for cell extraction, and stained with SYBR Green I (Molecular Probes Inc., Eugene, United States). SYBR Green I specifically binds to double-stranded DNA, serving as an indicator for intact cells (Zipper et al., 2004). Total cells were counted using a DM2000 epifluorescence microscope (Leica Biosystems, Nanterre, France), as previously described (Kallmeyer et al., 2008).

For DNA extraction, the whole-round core samples stored in N₂-flushed, heat-sealed aluminum bags were aliquoted in the home lab anaerobic chamber. Any sample showing signs of oxidation was discarded. Aliquots of ca. 20 g of sediment were subsampled using sterile cut-off syringes, re-packed under N₂ protective atmosphere, and stored at −80 °C until extraction. For 16S rRNA gene sequencing, we applied a depth-titered DNA extraction workflow, adapted from different commercial kits, as previously described (Ruiz-Blas et al., 2024, 2025). From 0 to 50 cmblf, we extracted DNA from 1 g using the GeneMATRIX Environmental DNA and RNA Purification Kit (EURx^®^, Gdánsk, Poland). From 1 to 5 mblf, we extracted DNA from 2 g using the DNeasy PowerSoil Kit from Qiagen (Hilden, Germany). From 5 to 15 mblf, we extracted DNA from 10 g using the DNeasy PowerMax Soil Kit from Qiagen. The final DNA elutions were concentrated from 5 mL into 200 μL, using Amicon Ultra Centrifugal Filters of 30 kDa (Merck KGaA, Darmstadt, Germany) and diluted 10-fold for PCR amplification.

Shotgun metagenomes were generated from selected horizons within the upper 15 mblf, using the same DNA extracts. Within the upper 50 cmblf, we used the GeneMATRIX Environmental DNA and RNA Purification Kit from ca. 300 mg of sediment split into two reactions. At 136, 245, and 555.5 cmblf, we used the DNeasy PowerSoil Kit with 6 to 8 parallel reactions of 1 g sediment each and pooled the final elutions. At 950 cmblf, we used two reactions of the DNeasy PowerMax Soil Kit for an initial amount of 20 g of sediment. DNA extracts were subsequently concentrated using Amicon Ultra Centrifugal Filters of 3 kDa (Merck KGaA) and purified using the MONARCH^®^ Genomic DNA Purification Kit (NEB, Massachusetts, United States) with an additional wash step.

### 2.6 Barcoded 16S rRNA gene libraries, Illumina sequencing and read processing

Partial 16S rRNA genes were amplified with barcoded primers 515F (5′-GTG TGY CAG CMG CCG CGG TAA-3′) and 806R (5′-CCG GAC TAC NVG GGT WTC TAA T-3′), purified using the HighPrep PCR Clean-up system (MagBio Genomics Inc., Gaithersburg, United States) and pooled, adding 20 ng of each cleaned PCR product per sample. The samples from the uppermost 50 cmblf were sequenced on a NovaSeq Illumina platform (Illumina, San Diego, United States), yielding 2 × 250 bps-long reads (Novogene GmbH, Munich, Germany), whereas the samples from 1 to 15 mblf were sequenced on an HiSeq Illumina platform with MiSeq v.3 chemistry, yielding 2 × 300 bps-long reads (Eurofins Genomics, Ebersberg, Germany). Reads were demultiplexed with Cutadapt v. 3.5 (Martin, 2011). Amplicon sequence variants (ASVs) were inferred with DADA2 v. 1.20 in R v. 4.1 (Callahan et al., 2016). Taxonomy was assigned against the SILVA SSU database 138.1 (Quast et al., 2013). ASVs matching chloroplasts, mitochondria, and singletons were removed. Mock communities, extraction blanks, and PCR negatives were processed and sequenced in parallel as previously reported (Ruiz-Blas et al., 2025).

A canonical correspondence analysis (CCA) was performed with 27 standardized explanatory variables and computed with the 2000 most abundant ASVs normalized to the total number of reads per sample, using Past v. 4.03 (Hammer et al., 2001). Significance of canonical axes was tested via permutation (*N* = 999).

### 2.7 Metagenome sequencing and read processing

Metagenomic libraries were prepared with the Nextera XT DNA Library Prep Kit (Illumina) and sequenced at CeGaT GmbH (Tübingen, Germany) on a NovaSeq 6,000 Illumina platform, yielding 2 × 150 bps-long reads and targeting 50 million read pairs per sample. Demultiplexing was performed using bcl2fastq v2.20. Adapters were trimmed with Skewer v. 0.2.2 and read quality-check (QC) assessed with FastQC v.0.11.5. QC reads were mapped to the SILVA SSU database 138.1 (Quast et al., 2013), using Bowtie 2 (Langmead and Salzberg, 2012). In addition, two intracellular DNA extracts previously isolated from 32 cmblf, (Vuillemin et al., 2018) applying a protocol tailored for separate retrieval of the intra- and extracellular DNA fractions (Vuillemin et al., 2017), were sent to the DOE Joint Genome Institute (JGI, Walnut Creek, CA) for metagenomic sequencing as part of the Microbial Dark Matter project (proposal ID: 1477). The corresponding raw sequencing datasets were downloaded, using sratoolkit v. 3.0.6, and processed alongside to leverage MAG quality. The 8 metagenomes from the gravity core were combined and processed as a single sample in parallel with the four metagenomes from the hydraulic core as to retain per-sample abundance estimates.

Metagenomic reads were processed with ATLAS v. 2.1.0 (Kieser et al., 2020), which uses quality control reads from BBTools to execute the following pipeline: metaSPAdes v. 3.11.1 for assembly, Prodigal v. 2.6.3 for extraction of open-reading frames (ORFs), eggNOG-mapper v. 2.1 for functional annotation, and binning with MetaBAT2 v. 2.1.5 and MaxBin v. 2.2.7 into metagenome-assembled genomes (MAGs), followed by dereplication with DAS Tool v. 1.1.6 (Supplementary references). MAGs were taxonomically assigned using GTDB-Tk against the GTDB database R202 v. 2.1.1 (Parks et al., 2022). In total, 101 MAGs were recovered, comprising 49 archaeal and 52 bacterial MAGs. Only MAGs displaying completeness ≥70% and contamination ≤10% were considered good-quality and selected for downstream analysis. In addition, phylogenetic analysis of the MAG taxonomy was performed by concatenating 16 ribosomal protein markers, using open scripts and protocols (Graham and Tully, 2018), implementing the closest representative MAGs available from the GTDB database (Parks et al., 2022) as references. Calculated tree distances were visualized using iTOL v. 6 (Letunic and Bork, 2024).

ORF functional predictions based on eggNOG were verified, performing BLASTp against the large aggregated MetaProt database (Orsi, 2020), using DIAMOND (Buchfink et al., 2015). Functional marker genes were identified on ORFs predicted from 81 good-quality MAGs only. Fe-related genes were identified using curated Hidden Markov Models implemented in the FeGenie pipeline (Garber et al., 2020), with default parameters. Targeted ORFs involved in dissimilatory and assimilatory sulfur metabolism included the enzymes *sat I-II*, *APS-kn*, *aprAB*, *dsrAB*, *dsrC*, *dsrE*, *asrA*, *psrA/phsB*, and *hyd I-II*, with *sudAB*, *cysJ*, *dmso*, *SOR*, *suox*, and *tauD* (Vuillemin et al., 2022). Targeted ORFs involved in dissimilatory nitrogen metabolism included the enzymes *nar*, *nap*, *nir*, *nor*, *nos*, and *nrf* (Vuillemin, 2023). Functional marker genes were selected according to the KEGG pathways of sulfur (i.e., map 00920) and nitrogen (i.e., map 00910) metabolisms. All names of enzymes and gene abbreviations are available as supplement (Supplementary Table S1).

Further phylogenetic analysis of the conserved amino acid alignments of ORFs encoding subunits of the respiratory (*nar*) and periplasmic (*nap*) nitrate reductase, dissimilatory sulfite reductase (*dsr*), and methyl-coenzyme M reductase (*mcr*) was conducted in SeaView v. 5.0.5 (Gouy et al., 2010). Conserved regions of the alignments were selected using Gblocks with the following settings: allowing for smaller final blocks, gap positions within the final blocks and less strict flanking positions. Phylogenetic trees were computed using PhyLM maximum likelihood (Guindon et al., 2010), with BLOSUM62 as the evolutionary model and 100 bootstrap replicates, and visualized using iTOL (Letunic and Bork, 2024).

## 3 Results

### 3.1 Bulk sediment reflects redox fluctuations and deposition of Fe inflows

**Figure 2:**
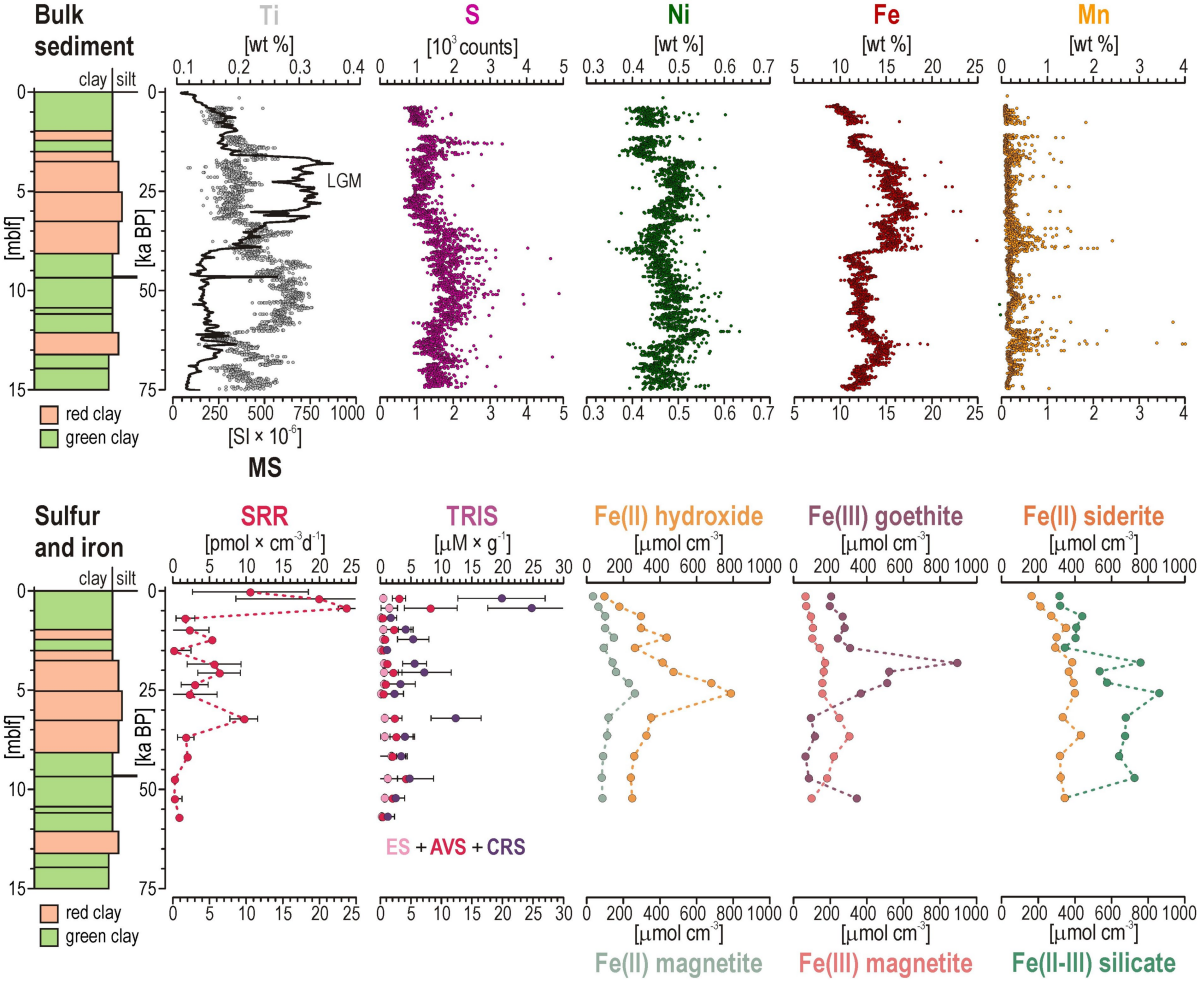
Bulk sediment geochemistry, sulfur speciation, and mineralogy of reactive iron in the 15 m-long sediment interval. (Top) From left to right: Downcore profiles for magnetic susceptibility (MS), bulk Ti, S, Ni, Fe, and Mn sediment content. The interval, in which MS values increase (~3 to 8 mblf), corresponds to the Last Glacial Maximum (LGM). (Bottom) From left to right: Depth-resolved sulfate reduction rates (SRR); total reduced inorganic sulfur (TRIS) divided into elemental sulfur (ES), acid volatile sulfide (AVS), and chromium reducible sulfide (CRS) fractions; and reactive iron phases and speciation corresponding to Fe(II) hydroxide, Fe(II) magnetite, Fe(III) goethite, Fe(III) magnetite, Fe(II) siderite, and Fe(II-III) silicate. Age calibration in kilo annum before present (Y-axis in ka BP) is based on Ulfers et al. (2021).

X-ray fluorescence core scanning profiles confirmed the ferruginous nature of Lake Towuti deposits, with Fe dominating the composition of bulk sediments throughout the upper 15 mblf (Figure 2). Fe concentrations are usually high (6–18 wt %), with little fluctuations apart from clear maximal values (~20 wt %) in an interval mostly composed of reddish clay (ca. 4–7 mblf). This interval corresponds to the Last Glacial Maximum (LGM), a climatic period during which lake levels were lower and the sediment–water interface likely oxygenated (Costa et al., 2015; Russell et al., 2020). The LGM interval is characterized by high magnetic susceptibility (MS) and Fe content due to abundant magnetite alongside other Fe(III) oxides and siderite, while Ti content is higher below the LGM interval. Together, the Fe and Ti contents reflect changes in sediment type related to the forced progradation of the Mahalona River delta during lake lowstand (Morlock et al., 2021). The S profile (not weight calibrated) shows rather low content and reveals generally slightly higher values in green clay consistent with organic matter content (Vuillemin et al., 2023b). The Ni profile broadly co-varies with Fe, reflecting a shared lateritic source of Ni-bearing Fe (oxyhydr)oxides. In addition, sporadic peaks in Fe, S and Ni tend to co-occur, suggesting authigenic formation of millerite (Supplementary Figure S1). The Mn profile presents discontinuous peaks, corresponding with the incorporation of dissolved Mn^2+^ from pore water into siderite (Vuillemin et al., 2023b).

### 3.2 Sulfate reduction rates and reduced sulfur species precipitation in sediment

Sulfate reduction rates in surface sediment are about 10–25 pmol × cm^−3^ × d^−1^ within the uppermost few centimeters, with values dropping close to detection limit at ca. 1 mblf (Figure 2). This sharp decrease coincides with pore water SO_4_^2−^ depletion in the same interval. Low but measurable sulfate reduction activity persists down to about 12 mblf. Consistent with low sulfate reduction rates, total reduced inorganic sulfur concentrations in the sediment are uniformly low, and mostly found as chromium-reducible sulfide (CRS) with minor acid volatile sulfide (AVS). Maximum values are found in the upper mblf, but less pronounced peaks also occur around 6 and 11 mblf. This corresponds to the LGM interval in which bulk S and pore-water SO_4_^2−^ subsequently increase again. We infer that the chromium-reducible sulfide pool, usually assumed to represent disulfides (e.g., pyrite), may derive from millerite (NiS) instead (Supplementary Figure S1), as nickel is more chalcophile than iron. This further implies that any HS^−^ produced during sulfate reduction can quickly react with pore water Ni and Fe and be sequestrated as millerite.

### 3.3 Fe speciation highlights increased redox cycling during the LGM

Sequential extractions show that reactive Fe phases vary with depth and track sediment inflows and past redox conditions (Sheppard et al., 2021), revealed by an increased content of Fe oxides in the LGM red-clay interval (Figure 2). Easily reducible Fe(II) (0.5 N HCl-extractable) increases from about 100 μmol × cm^−3^ near the surface to about 750–800 μmol × cm^−3^ at 5–6 mblf (Figure 2), reflecting complete microbial reduction of ferrihydrite. Right above, concentrations in crystalline Fe(III) oxide (sodium dithionite-extractable) exhibit an increase from 200 to 800 μmol × cm^−3^ between 4 and 6 mblf, indicating a substantial Fe(III) reservoir, likely preserved as goethite (a FeOOH). The co-occurrence of a substantial reactive Fe(II) pool with abundant but less reactive Fe(III) oxides may reflect intensified Fe redox cycling during the LGM, namely increased lateritic inflows to an oxic lake floor and subsequent microbial diagenesis (Sheppard et al., 2019; Russell et al., 2020). The sediment content in magnetite (ammonium oxalate-extractable) also shows slightly increasing values down to 5–8 mblf. Discrepancies between the two profiles (i.e., magnetite-extracted Fe^2+^Fe^3+^_2_O_4_) may capture microbial reduction of less reactive mixed-valence Fe phases. In comparison, the sediment Fe content corresponding to siderite (sodium acetate-extractable) increases from 200 to 400 μmol × cm^−3^ in the upper 3 mblf, showing that siderite (FeCO_3_) accumulates as an early diagenetic product of microbial reduction (Vuillemin et al., 2019). The overall increase in silicate-related Fe (6 N HCl extractable) down to 4 mblf (300 to 900 μmol × cm^−3^) corresponds with increased siliciclastic sedimentation during the LGM (Morlock et al., 2021).

Altogether, the LGM interval seems to have experienced increased Fe inflows followed by intense microbial Fe cycling in the vicinity of an oxygenated sediment–water interface. The source-to-sink processes behind the peaks in Fe(II) hydroxide and Fe(III) goethite observed between 4 and 7 mblf (Figure 2) were interpreted as resulting from an increased flux of reactive Fe oxides, lake mixing and bottom water oxygenation (Russell et al., 2020; Sheppard et al., 2021), followed by microbial reductive dissolution and diagenetic redistribution of iron into authigenic phases, e.g., magnetite, siderite, vivianite (Vuillemin et al., 2020, 2022).

### 3.4 Pore water profiles

**Figure 3:**
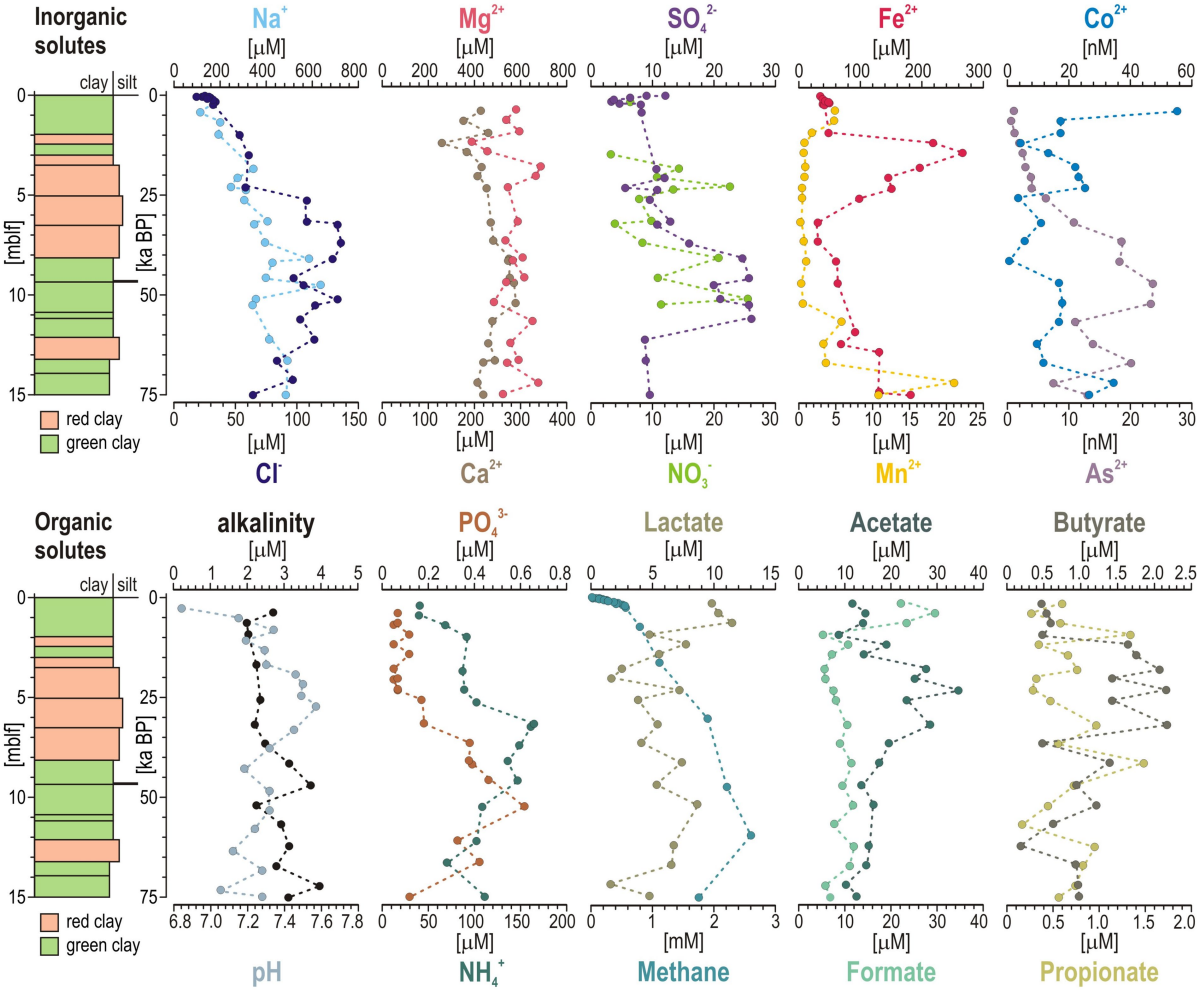
Pore water geochemistry in the 15 m-long sediment interval. (Top) From left to right: Downcore profiles for pore water inorganic solutes, i.e., Na^+^, Cl^−^, Mg^2+^, Ca^2+^, SO_4_^2−^, NO_3_^−^, Fe^2+^, Mn^2+^, Co^2+^, and As^2+^. (Bottom) From left to right: Downcore profiles for pore water organic solutes related to organic matter breakdown, i.e., alkalinity, pH, PO_4_^3−^, NH_4_^+^, methane, and volatile fatty acids, namely lactate, formate, acetate, propionate, and butyrate. Age calibration is based on Ulfers et al. (2021).

SO_4_^2−^ concentrations are ca. 12 μM at the sediment–water interface and drop close to detection limit (2 μM) in the upper mblf (Figure 3), delimiting the active SO_4_^2−^ reduction zone to shallow sediment depths. SO_4_^2−^ concentrations increase again between 8 and 12 mblf in the LGM interval, coinciding with small peaks in total reduced inorganic sulfur and bulk sediment S concentrations (Figure 2). NO_3_^−^ concentrations are below detection in the water column (i.e., 4 μM) and thus nitrate/nitrate is mostly absent in pore water. However, discontinuous peaks (<30 μM) occur between 3 and 10 mblf, indicating transient availability of nitrate as an oxidant within otherwise reduced sediment strata (Figure 3). Altogether, an increased availability in SO_4_^2−^ and NO_3_^−^ as electron acceptors is observed in the LGM interval. Similarly, the increase in Na^+^ and Cl^−^ pore water profiles is consistent with a salinity increase and lake evaporation during that time (Costa et al., 2015). Dissolved Fe^2+^ sharply increases in pore water to about 250–280 μM at 3 mblf, further declining down to 15 mblf, highlighting the interval in which dissimilatory Fe(III) reduction may be microbially active. The subsequent decrease in dissolved Fe^2+^ concentrations indicates precipitation of authigenic phases (Vuillemin et al., 2023b). After an initial increase in the upper 2 mblf consistent with early microbial reduction, Mn^2+^ concentrations are consistently low in pore water as siderite acts as the main sink (Figures 2, 3), exhibiting a single peak around 14 mblf. Co^2+^ concentrations exhibit a similar trend to the one of Mn^2+^, whereas As^2+^ increases in pore water with increasing reducing conditions of the sediment with depth (Figure 3). Pore water Mg^2+^ (ca. 600 μM) and Ca^2+^ (ca. 200 μM) exhibit steady profiles consistent with the dissolution of mafic inputs (e.g., serpentinite).

Organic solutes are very low in near-surface sediments (Figure 3). For instance, PO_4_^3−^ and NH_4_^+^ gradually increase down to 6–10 mblf, and subsequently decrease slightly, tracing organic matter mineralization (Figure 3). Volatile fatty acids are produced during fermentation and consumed during remineralization to methane (Glombitza et al., 2019), resulting in their turnover in pore water (Figure 3). Lactate, formate and butyrate are mostly produced during acidogenesis, and their respective pore water concentrations successively increase in near-surface sediment, followed by their oxidation during acetogenesis. This second step results in acetate accumulating in pore water around 5 mblf. As remineralization of volatile fatty acids proceeds, methane is continuously produced as the end product and concentrations gradually increase to 3 mM from the sediment–water interface down to 15 mblf. Accordingly, pore water alkalinity increases gradually with depth, buffering pH values between 7 and 7.5. Altogether, pore water profiles for organic solutes are consistent with processes of stepwise organic matter degradation (McInerney et al., 2009).

### 3.5 Microbial cell abundances

**Figure 4:**
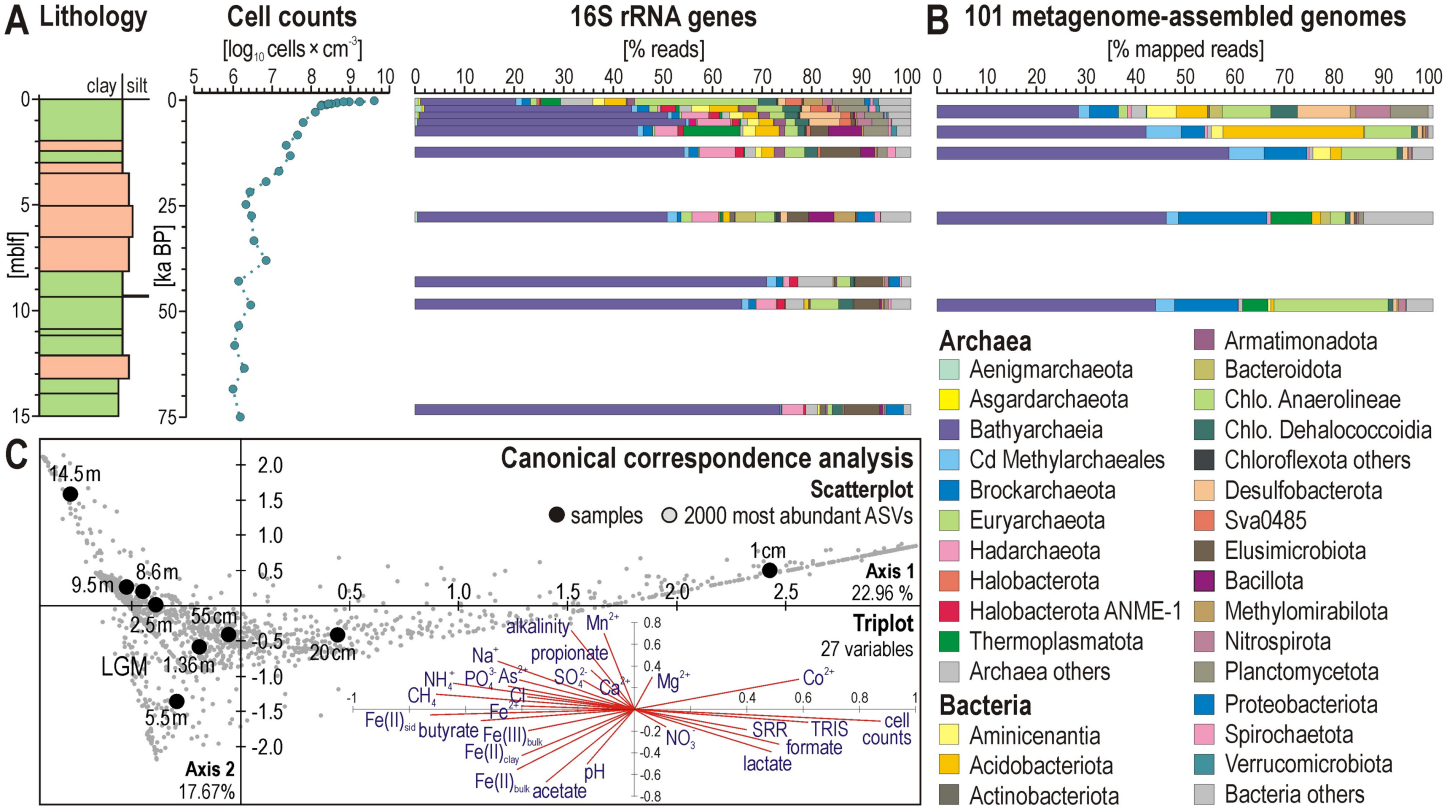
Microbial cell abundance, 16S rRNA genes, and metagenome-assembled genomes. **(A)** Total cell counts and relative abundances of 16S rRNA gene reads at the phylum/class level. **(B)** Relative taxonomic abundances of metagenome-assembled genomes (MAGs) based on the number of metagenomic reads mapped to each of the 101 MAGs at the phylum/class level. **(C)** Scatterplot of the canonical correspondence analysis (CCA) computed with the 2000 most abundant amplicon sequence variants (ASVs) obtained from 16S rRNA gene libraries, and triplot computed with 27 explanatory variables.

Microbial cell densities (Figure 4A) are highest underneath the sediment–water interface (~9.5 log_10_ cells × cm^−3^), reflecting a greater availability in labile organic matter in upper sediments and electron acceptors in the pore water (Figure 3). In the uppermost 10 cmblf, cell counts steeply drop, reaching 7.6 log_10_ cells × cm^−3^ at 1 mblf, followed by a gradual decline to 6.3 log_10_ cells × cm^−3^ down to 5 mblf. Below this depth, values remain constant, indicating a low population turnover but persistent microbial population in deep sediments. The steep decrease in cell density observed for shallow sediments coincides with the rapid depletion of respiratory electron acceptors (mainly Fe^3+^, and SO_4_^2−^) under ferruginous conditions and the transition from sulfate reduction to fermentation in the sediment sequence (Ruiz-Blas et al., 2024).

### 3.6 Selective assembly of 16S rRNA genes in the subsurface

Bioinformatic treatment of 16S rRNA gene sequences from 23 discrete samples resulted in 279,048 processed reads assigned to 4,851 curated ASVs, of which 2,202 ASVs (~45%) were identified as Archaea and 2,649 ASVs (~55%) as Bacteria (Supplementary Data). Archaeal populations are predominantly represented by the phyla/classes Bathyarchaeia, Hadarchaeota, and Nitrososphaeria, whereas most abundant bacterial phyla correspond to Aminicenantia, Chloroflexota (i.e., classes Anaerolineae and Dehalococcoidia), Desulfobacterota, and Elusimicrobiota (Figure 4A). Chloroflexota account for ca. 30% of total 16S rRNA reads at the sediment–water interface. Despite an initial ~10% decrease in the upper 0.5 mblf, this phylum remains predominant in the taxonomic assemblage with depth, stabilizing at <10% down to 10 mblf. Desulfobacterota are also relatively abundant at the sediment–water interface (ca. 10–15%), but decrease consistently with sulfate reduction rates (Figures 2, 4). Bathyarchaeia consistently increase from 20 to 40% of all reads in the upper 0.5 mblf, depth at which they account for the majority of the taxonomic assemblage. Bathyarchaeia become even more abundant, accounting for >70% of 16S rRNA reads at 10 mblf and below, clearly prevailing in the taxonomic diversity of deep populations (Figure 4).

Based on the CCA analysis (Figure 4C), variations in beta diversity, alongside cell densities, sulfate reduction rates and pore water geochemistry, can be broadly interpreted as the distribution of abundant phyla into functional guilds reflecting different metabolic roles due to substrate depletion with sediment depth (e.g., iron reducers, sulfate reducers, fermenters, methanogens, homoacetogens). As cell counts and sulfate reduction rates decrease in the upper mblf, the taxonomic diversity shifts from bacteria-dominated near the surface to archaeal-dominated populations, reflecting the transition from the sulfate reduction to the fermentative zone. Axis 1, which accounts for 22.96% of the explained variance, partly confirms the correlation between taxonomic assemblages and geochemical evolution of pore water during reductive diagenesis. The main explanatory variables behaving as end members in the triplot can be interpreted as a decrease in microbial populations with depth as they transit from the sulfate reduction zone into the fermentation zone (right, i.e., cell counts, TRIS, SRR, DIC, formate, lactate), ending with methanogenesis and an accumulation of reduced solutes in pore water (left, i.e., Fe^2+^, PO_4_^3−^, NH_4_^+^, As^2+^, butyrate, CH_4_). Axis 2 accounts for 17.67% of the explained variance. The scatterplot (Figure 4C) mostly emphasizes a specific sample at 5.5 mblf in the LGM interval and the lowermost sample at 14.5 mblf. The corresponding explanatory variables in the triplot highlight the increased presence of sediment iron oxides and pore water acetate for the former sample (i.e., Fe(II), Fe(III), pH, acetate) and the accumulation of reduced pore water solutes for the latter sample (i.e., Mn^2+^, As^2+^, NH_4_^+^, propionate, alkalinity).

### 3.7 Taxonomy and predicted functions of subsurface community metagenomes

Along the 15-m-long record, the abundance of reads mapped to MAGs is mostly consistent with those of 16S rRNA genes, highlighting the prevalence of Bathyarchaeia below 1 mblf, which recruit > 40% of the total metagenomic reads (Figure 4B).

*De novo* assembly of metagenomic reads yielded 101 MAGs (Supplementary Tables S2, S3; Supplementary Data) of which 81 were considered good-quality (>70% completeness; <10% contamination) and selected for downstream analysis (Supplementary Figure S2). In total, 38 archaeal and 43 bacterial MAGs were processed for phylogenomic analysis and characterization of specific metabolic features of their genetic content. Phylogenetic analysis of concatenated ribosomal proteins confirmed taxonomic assignment of good-quality MAGs to: 24 Bathyarchaeia, 5 Nitrososphaerales, 1 Methanomethylicia, 1 Methanobacteriota, 2 Thermoplasmatota, 2 Halobacteriota, and 3 Brockarchaeota among Archaea (detailed in Supplementary Figure S3); and 8 Desulfobacterota, 7 Acidobacteriota, 6 Nitrospirota, 11 Chloroflexota, 1 Elusimicrobiota, 3 Planctomycetota, 2 Bacteroidota, 1 Methylomirabilota, 1 Bacillota, and 2 candidate phyla for Bacteria (detailed in Supplementary Figure S4).

**Figure 5:**
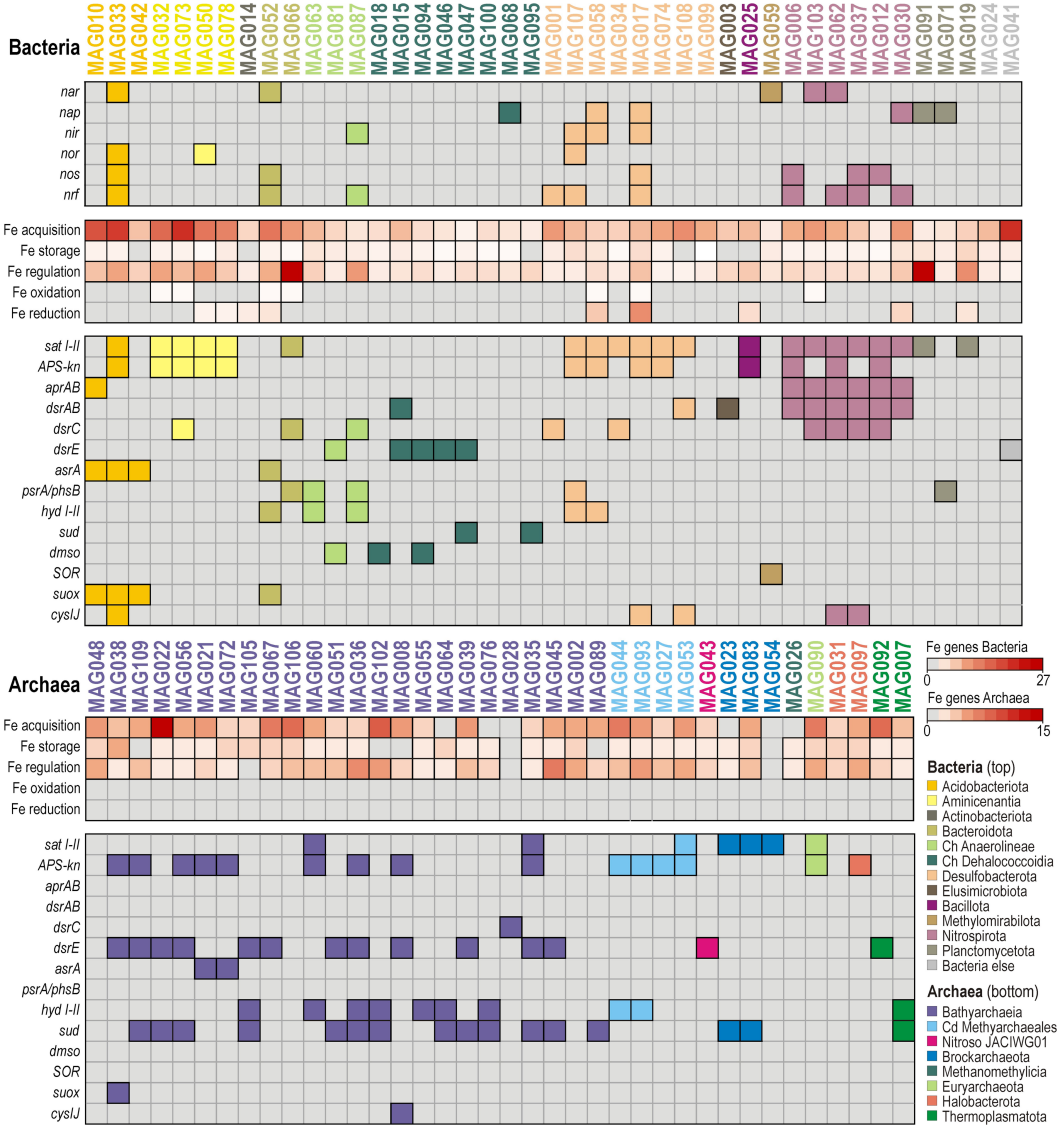
Repertoire of functional marker genes involved in redox processes of the nitrogen, iron, and sulfur biogeochemical cycle identified in good-quality metagenome-assembled genomes. Heatmap with presence/absence of specific functional marker genes in the genetic content of the 81 good-quality (>70% completeness; <10% contamination) metagenome-assembled genomes (MAGs) obtained in this study. Columns represent individual bacterial (top) and archaeal (bottom) MAGs, and rows indicate presence/absence of functional markers genes in the corresponding MAGs. The abundances of iron-related genetic functions are based on the FeGenie pipeline (Garber et al., 2020). Names of enzymes and gene abbreviations are available as supplement (Supplementary Table S1).

Functional marker genes relevant to denitrification and dissimilatory nitrate/nitrite reduction to ammonium (DNRA) were identified in few bacterial MAGs (Figure 5). Those exhibiting consistent metabolic potential towards complete denitrification (*nar*, *nap*, *nir*, *nor*, *nos*) are found exclusively among the phyla Nitrospirota and Desulfobacteriota, otherwise metabolic potential for denitrification indicates a partial pathway (Planctomycetota, Methylomirabilota), and/or its stepwise partition (Bacteroidota, Chloroflexota), in combination with DNRA (*nrf*). Metabolic potential for anaerobic respiration of nitrogen species was not detected in archaeal MAGs. Phylogenetic analysis of *nar* and *nap* protein sequences further demonstrates that metabolic potential for nitrate reduction is present in *Geobacteraceae* (phylum Desulfobacteriota), Thermodesulfovibrionales (phylum Nitrospirota) and Rokubacteriales (phylum Methylomirabilota) (Supplementary Figure S5A).

Results of the FeGenie pipeline (Garber et al., 2020) indicate that metabolic potential for Fe acquisition, storage, and regulation is widespread across both archaeal and bacterial MAGs (Figure 5), whereas capacity for dissimilatory Fe reduction is phylogenetically restricted to MAGs among Acidobacteriota and Nitrospirota. Few additional hits to ORFs indicative of metabolic potential for Fe oxidation were detected in bacterial MAGs, namely in Desulfobacterota (2), Aminicenentia (4), Bacteroidota (2), and Thermodesulfovibrionia (1). These results indicate that cellular management of Fe is ubiquitous, whereas metabolic potential for Fe respiration is in comparison rather phylogenetically narrow. This suggests that FeRB, mostly among Aminicenantia and Desulfobacterota, could initially activate the turnover of reactive Fe phases in shallow sediments while most microorganisms subsequently mobilize, or re-incorporate, dissolved Fe ions through ancillary pathways (Garber et al., 2020).

Methanogens and anaerobic methanotrophs were identified via phylogenetic analysis of *mcrA-D* protein sequences (Supplementary Figure S5B), which confirmed the presence of the corresponding marker genes in Methanomassiliicoccales, Methyarchaeales (Ou et al., 2022), Methanomicrobiales and ANME-1).

Potential towards dissimilatory sulfate reduction (*aprAB*, *dsrAB*) is uncommon among our 81 good-quality MAGs (Supplementary Figure S6), and is exclusively found in Desulfobacterota and Nitrospirota, specifically in the class Thermodesulfovibrionia. Microorganisms related to the corresponding MAGs (Figure 5) can thus respire sulfate, or sulfite, when available. However, such limited metabolic potential towards dissimilatory sulfate reduction is consistent with complete consumption of SO_4_^2−^ and rapidly declining sulfate reduction rates within the upper 2 mblf (Figure 2). Further potential for respiration of sulfur intermediates (*asr*, *psr/phs*, *hyd*) was irregularly distributed among Aminicenantia, Bacteroidota, Anaerolineae, Desulfobacterota and Bacilotta (Figure 5). In addition, metabolic potential to respire dimethyl sulfoxide anaerobically (*dmso*) was identified in MAGs assigned to diverse Chloroflexota. In contrast, MAGs of Bathyarchaeia consistently include ORFs encoding sulfhydrogenase (*hyd I-II*) and ferredoxin-reducing sulfide dehydrogenase (*sudAB*), with dissimilatory sulfite reductase specific subunits acting as sulfur carriers (*dsrC*, *dsrE*) (Venceslau et al., 2014; Löffler et al., 2020). Rather than a respiratory process, these enzymes can drive the reaction of elemental sulfur with sulfide to form polysulfides, thereby yielding electrons to reduce ferredoxin (Ruiz-Blas et al., 2024). Altogether, ORFs encoding the subunits *dsrC* and *dsrE*, acting as sulfur carriers (Venceslau et al., 2014; Löffler et al., 2020), are relatively common among all aforementioned MAGs (Figure 5), as detailed phylogenetic analysis of *dsr* protein sequences could confirm (Supplementary Figure S6).

Finally, potential for aerobic sulfite oxidation (*suox*) was identified in MAGs assigned to Acidobacteriota (i.e., Acidoferrales), whereas a single ORF encoding for aerobic sulfur disproportionation (*SOR*) was identified in one MAG (Methylomirabilota). ORFs indicative of oxidative assimilation of (in)organic sulfur (*tauD*, *SOX*) were not detected.

## 4 Discussion

### 4.1 Dissimilatory respiration toward sulfidogenic syntrophy via cryptic redox processes

**Figure 6:**
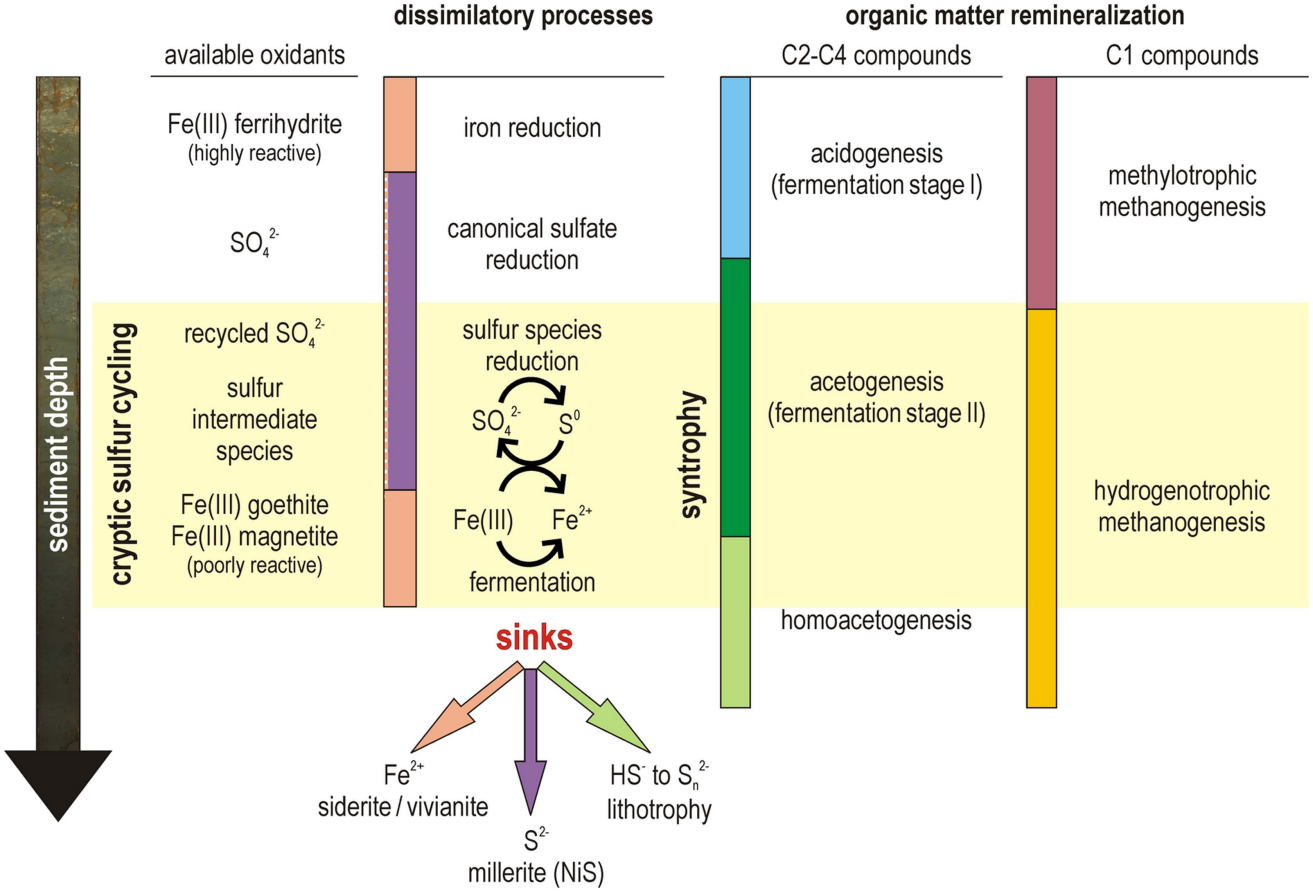
Metabolic transitions at the interface between dissimilatory processes and fermentative remineralization along the redox gradient developing with sediment depth. The interval of active cryptic sulfur cycling corresponds with syntrophic interactions with sulfate-reducing bacteria supported by fermentative reduction of iron oxides. While hydrogenotrophic methanogenesis is purely lithoautotrophic, homoacetogenesis involves mixotrophy on carbon sources combined with lithotrophy on sulfur and molecular hydrogen.

Beside pore water sulfate scarcity (<12 μM), Lake Towuti’s geochemical gradients deviate from the classic redox zonation by exhibiting high availability of stable Fe(III) oxides in its sediment (Figure 2). While SRB survive under decreasing availability of labile organic substrates by engaging in syntrophic partnerships with fermenters, i.e., sulfidogenic-acetogenic syntrophy (Sieber et al., 2012), the same syntrophic fermenters also shuttle electrons via poorly soluble Fe(III) minerals (Shi et al., 2016), thereby indirectly acting as FeRB (Kappler et al., 2021). This suggests that microbial sulfate and iron reduction may overlap and proceed in parallel through syntrophic partnerships (Figure 6). Such syntrophic consortia are well-documented in anoxic environments (McInerney et al., 2009; Sieber et al., 2012) and were expected to be active in ferruginous settings (Vuillemin et al., 2018).

Despite monosulfide sequestration as authigenic millerite (Supplementary Figure S1), sulfate reduction rates demonstrate that sulfidogenic activities still occur at very low SO_4_^2−^ concentrations (~10 mM) between 3 and 10 mblf, while pore water profiles of volatile fatty acids reveal efficient turnover with a transient increase in acetate concentrations (Figures 2, 3). The slight increase in pore water SO_4_^2−^ concentrations (i.e., from 10 to 26 mM) alongside the presence of Fe(III) oxides (i.e., goethite, magnetite) delineate a sedimentary interval in which a cryptic sulfur cycle appears to be actively maintained by microbial consortia. Metagenomic analysis revealed that sulfate uptake (*sat I-II*, *APS-kn*), dissimilatory sulfate reduction (*aprAB*, *dsrAB*) and related sulfidogenic processes (*asrA*, *psrA/phsB*, *hyd I-II*) could be performed by several taxa (Figure 5). Consistent with known sulfate-reducing lineages (Castro et al., 2000), MAGs holding key genes predicted to function in canonical sulfate reduction were predominantly affiliated with Desulfobacterota, Nitrospirota (class Thermodesulfovibrionia) and Bacillota (Supplementary Figure S6), whereas reduced sulfur and organosulfur (*dmso*) species could be respired by diverse Chloroflexota (Figure 5). Dissimilatory iron reduction was concomitantly present in MAGs assigned mostly to Aminicenantia, but also detected in a few additional MAGs (i.e., *Geobacterales*, *Symbiobacterales*, *Nitrospirales*, *Sedimentisphaerales*). Although reactive Fe(III) oxyhydroxides (i.e., ferrihydrite) were likely entirely reduced in the water column (Bauer et al., 2020) and upper centimeters of sediment (Friese et al., 2021), these potential FeRB could still use less reactive structural Fe(III) from goethite and magnetite as electron acceptor (Shi et al., 2016; Kappler et al., 2021) via extracellular electron transfer with c-type cytochromes (i.e., outer-membrane *omc* complex). Furthermore, FeRB and SRB can adjust their metabolisms according to available substrates and electron acceptors, evolving as secondary fermenters when sulfate deficiency prevents complete oxidation of organic substrates (Plugge et al., 2011). In such case, FeRB and SRB essentially scavenge volatile fatty acids and molecular hydrogen to ensure energy-yielding reactions in association with other syntrophs (Figure 6) among Desulfobacterota (*Smithellaceae, Syntrophaceae*) and fermenters (e.g., Elusimicrobiota, Chloroflexota).

In addition to sulfate and iron respiration, our metagenomic data indicate a potential respiratory contribution from the nitrogen cycle (Figure 5), which seems to include nitrate or nitrite-dependent anaerobic oxidation of reduced sulfur and iron, using acetate as organic substrate (Cardoso et al., 2006; Picardal, 2012). Between 3 and 10 mblf, pore waters contain low, discontinuous NO_3_^−^ peaks (Figure 3) mirrored by a patchy but diagnostic set of denitrification/DNRA-related functional genes identified in specific bacterial MAGs (Figure 5; Supplementary Figure S5). Among these, some Desulfobacterota (incl. Geobacterales) hold metabolic potential for either complete or partitioned denitrification (*nap*, *nir, nor, nos*), whereas the Nitrospirota (incl. Thermodesulfovibrionales) appear capable of combining partial denitrification (*nar*) with DNRA (*nrf*). While these sparse metabolic traits are consistent with NO_3_^−^/ NO_2_^−^ respiration (Kuypers et al., 2018), they also suggest a secondary role in a cryptic iron–sulfur cycle via nitrate-dependent anaerobic oxidation (Vuillemin et al., 2018; Berg et al., 2019). Similar metabolic potential related to nitrate reduction coupled with aerobic oxidation of sulfur (*suox*) and disproportionation (*SOR*) was detected among MAGs assigned to Acidobacteriota, Bacteroidota and Methylomirabilota (Figure 5).

In parallel to cryptic redox processes, stepwise fermentation of organic matter to methane proceeds gradually (Figure 3), with production and consumption of volatile fatty acids as intermediates (McInerney et al., 2009; Sieber et al., 2012). Taxonomic and functional profiling of populations with depth (Figure 4) evidences a consortium of versatile FeRB and SRB, diverse methanogens (Supplementary Figures S3, S5), and abundant homoacetogens including Chloroflexota (Vuillemin et al., 2024) and mostly Bathyarchaeia (Ruiz-Blas et al., 2024). The co-occurrence of two MAGs assigned to ANME-1b throughout the sediment sequence (Figure 4; Supplementary Figure S5B) raises the question of whether metabolic activity towards cryptic production (Beulig et al., 2019) and/or anaerobic oxidation of methane (AOM) with an oxidant other than sulfate (Beal et al., 2009) is present, as typically performed by the ANME-2 clades (Milucka et al., 2012). Although partnership with SRB via direct interspecies electron transfer has been shown for the ANME-1c clade (Benito Merino et al., 2022), the absence of multiheme cytochromes in the ANME-1b clade suggests a putative methanogenic metabolism (Laso-Pérez et al., 2023).

Thus, despite a scarcity of sulfate and limited reactivity of Fe(III) mineral substrates, Lake Towuti microbial communities residing in the upper 10 mblf appear to actively exploit any available pore water electron acceptor and donor to achieve cooperative metabolic strategies across functional guilds (Figure 6). Below 10 mblf, organic solutes are close to depletion while methane accumulates as the end-product of remineralization (Figure 3).

### 4.2 Cryptic sulfur cycle and homoacetogenesis as final electron sink

In Lake Towuti sediments, any sulfide produced initially during sulfate reduction is removed, precipitating with pore water Ni^2+^ as millerite (Supplementary Figure S1). This keeps HS^−^ concentrations in the pore water below detection, and transfers HS^−^ into the chromium-reducible sulfide pool (Figure 2). Below the upper mblf where sulfate reduction rates drop (Figure 2), pore water Fe^2+^ precipitates with biogenic DIC as siderite, and deeper with PO_4_^3−^ as vivianite (Vuillemin et al., 2019, 2020). Yet, low but sustained sulfate reduction occurs in mineral Fe(III)-rich sediment horizons, spatially confining the presence of a detectable cryptic sulfur cycle potentially driven by iron (Figure 6). Furthermore, the presence of NO_3_^−^ in pore water can apparently lead to some anaerobic oxidation of dissolved Fe^2+^ or HS^−^, and any intermediate sulfur species (i.e., SO_3_^2−^, S_2_O_3_^2−^, S_2_O_6_^2−^, S_X_^0^) produced in this process are being rapidly scavenged. However, Fe(III) minerals could also react abiotically with sulfide to form polysulfide, thereby mimicking some residual sulfate reduction activity (Hansel et al., 2015).

Some marker genes indicative of biotic cycling of intermediate sulfur species were detected (Figure 5). Many MAGs included ORFs coding for *dsrC* and *dsrE*, which act as intracellular sulfur carriers (Venceslau et al., 2014; Löffler et al., 2020), differently combined with respiratory functions (Zhou et al., 2025), i.e., sulfite (*asr*), thiosulfate (*phs/psr*), polysulfide and elemental sulfur (*hyd*). Minor respiratory reduction of sulfur intermediates could be predicted for Desulfobacterota (incl. Desulfuromonadales, Geobacterales), Acidobacteriota and Chlorofleoxta, phyla which all exhibited some metabolic versatility in the use of electron acceptors (Figure 5), e.g., nitrate/nitrite, ferric iron, sulfate and dimethyl sulfoxide. In contrast, multiple fermentative Bathyarchaeia were clearly predicted to encode sulfhydrogenase (*hyd I-II*) with reversible sulfide dehydrogenase (*sudAB*), which provide them the metabolic capacity to harness electrons from sulfide via polysulfide (Ruiz-Blas et al., 2024). Instead of elemental sulfur respiration (Ma and Adams, 1994), these electrons are bifurcated to reduce ferredoxin and drive homoacetogenesis via a complete methyl-branch WLP for energy production and CO_2_ fixation (Hou et al., 2023). Autotrophic acetate synthesis provides a route to channel redox power into a single stable intermediate that can either accumulate, be utilized in syntrophic partnerships, or be incorporated anabolically into cellular biomass (Wolfe, 2005). By running the WLP in reverse, SRB scavenge acetate from pore water and mitigate the potential for product inhibition (Ragsdale and Pierce, 2008), enabling homoacetogens to drive the acetogenic reaction forward despite its relatively low energy yield.

The clear predominance of Bathyarchaeia among deep subsurface microbial communities and their predicted homoacetogenic lifestyle (Ruiz-Blas et al., 2024) underscore their capability to sustain slow carbon cycling in the energy-limited deep subsurface (Rodriguez et al., 2025; Ruiz-Blas et al., 2025). By breaking down complex organic substrates, Bathyarchaeia yield simple C1 compounds (Yu et al., 2023), which they then assimilate through homoacetogenesis (Feng et al., 2019; Hou et al., 2023). As an alternative to the use of molecular hydrogen, Bathyarchaeia harness redox power by reacting sulfide to polysulfide and bifurcating electrons to reduce ferredoxin (Ruiz-Blas et al., 2024). Their WLP further acts as terminal electron sink for redox balancing in fermentation, preventing a cessation of OM decomposition (Müller, 2003). Such lithotrophic lifestyle derived from sulfur “inorganic fermentation” gives Bathyarchaeia an additional competitive edge in substrate-limited settings.

Our study presents interesting analogies to ancient ferruginous systems (Lyons et al., 2024) in which early anaerobic microbial life was assumedly using dissimilatory reduction of insoluble Fe(III) oxides (Schröder et al., 2003) and redox-reactive sulfur species (Nitschke et al., 2013) coupled to the WLP via electron bifurcation (Weiss et al., 2016). Metagenomic predictions state that bacterial enzymes involved in redox cycling of sulfate and thiosulfate spread and diversified after the Great Oxidation Event (Mateos et al., 2023). Similarly, cryptic iron cycling would require mixed-valent iron minerals in the presence of an oxidant (Kappler and Bryce, 2017). Such cryptic interactions were observed in the LGM sediment interval which featured an oxygenated sediment–water interface, increased mineral Fe(III) flux and higher availability of pore water oxidants. In contrast, sulfur disproportionation with sulfide to form polysulfides represents an exergonic reaction under ferruginous conditions (Jørgensen, 2021) that autotrophic Archaea could biologically control to yield redox power on a pre-oxygenated Earth.

## 5 Conclusion

Our results show that the sediment subsurface of ferruginous Lake Towuti hosts a spatially confined cryptic sulfur cycle driven by a consortium of FeRB and SRB alongside syntrophic and lithotrophic (homo)acetogens. Sulfur disproportionation could in theory proceed via potential oxidants in the form of poorly reactive goethite and intermittent pore water nitrate. In parallel, Bathyarchaeia couple sulfur disproportionation via polysulfides to ferredoxin reduction in the WLP, thereby defining homoacetogenesis as the terminal electron sink in the deep subsurface.

As the autotrophic origin of life, the WLP is postulated to have arisen in hydrothermal environments that featured oases of bioavailable metals and reactive sulfur, contrasting with the prevalent redox state of the global ferruginous ocean. Lithotrophic homoacetogenesis would have preceded other dissimilatory sulfur metabolisms as it could be fueled by redox energy from sulfide via polysulfides. In comparison, cryptic sulfur cycling with other oxidants would require whisps of oxygen to precipitate mineral Fe(III) necessary to sulfur disproportionation and conversion of ammonium to nitrate. Consistent with the emergence of syntrophic interactions, modern dissimilatory metabolisms exhibited a high degree of versatility in supplying biogenic molecular hydrogen to SRB and methanogens facing substrate limitation.

To conclude, the stratigraphic record of Lake Towuti enabled to document geochemical signals, metagenomic predictions, and mineralogical fingerprints consistent with a cryptic sulfur cycle, providing constraints that are transferable from a present-day analogue to ancient ferruginous ecosystems.

## Data Availability

The datasets presented in this study can be found here: https://www.ebi.ac.uk/ena, accession PRJEB14484, PRJEB85713, PRJEB66721 and PRJEB98232; https://www.ncbi.nlm.nih.gov/, accession SRR5215464 and SRR5215466. The geochemical datasets (#861437, #908080, #934401) are publicly available from the PANGAEA® Data Publisher for Earth & Environmental Science (https://doi.pangaea.de/) under DOI no. 10.1594/PANGAEA.861437, 10.1594/PANGAEA.908080, and 10.1594/PANGAEA.934401.
