# Supplementary material for: Cryptic sulfur cycling in the deep biosphere of ferruginous Lake Towuti, Indonesia
Source: Front Microbiol. 2025 Dec 11;16:1725877. doi: 10.3389/fmicb.2025.1725877 (PMC12740246; doi:10.3389/fmicb.2025.1725877)
Supplement: Supplementary file 1 [file Data_Sheet_1.PDF]

Supplementary Material

1 Supplementary Figures

Supplementary Figure S1. Morphology and composition of authigenic (Fe,Ni)-sulfide minerals in Lake Towuti sediments.

Supplementary Figure S2. Determination of metagenome-assembled genomes (MAGs) quality.

Supplementary Figure S3. Phylogenetic tree based on 16 concatenated ribosomal protein markers for archaeal MAGs, with closest representative MAGs from the GTDB database as references.

Supplementary Figure S4. Phylogenetic tree based on 16 concatenates ribosomal protein markers for bacterial MAGs, with closest representative MAGs from the GTDB database as references.

Supplementary Figure S5. Phylogenetic trees of respiratory and periplasmic nitrate reductase and methyl-coenzyme M reductase gene proteins.

Supplementary Figure S6. Phylogenetic tree of dissimilatory sulfite reductase gene proteins.

2 Supplementary Tables

Supplementary Table S1. List of enzymatic names and abbreviations for functional marker genes predicted from *de novo* assembly

Supplementary Table S2. Metadata on the *de novo* assembly of metagenomic reads into contigs for the 5 libraries successfully sequenced.

Supplementary Table S3. Metadata on the *de novo* assembly of the 101 metagenome-assembled genomes obtained in this study.

3 Supplementary Data

Supplementary Data. Sequencing data and metadata as separate file (Supplementary Data.xlsx)

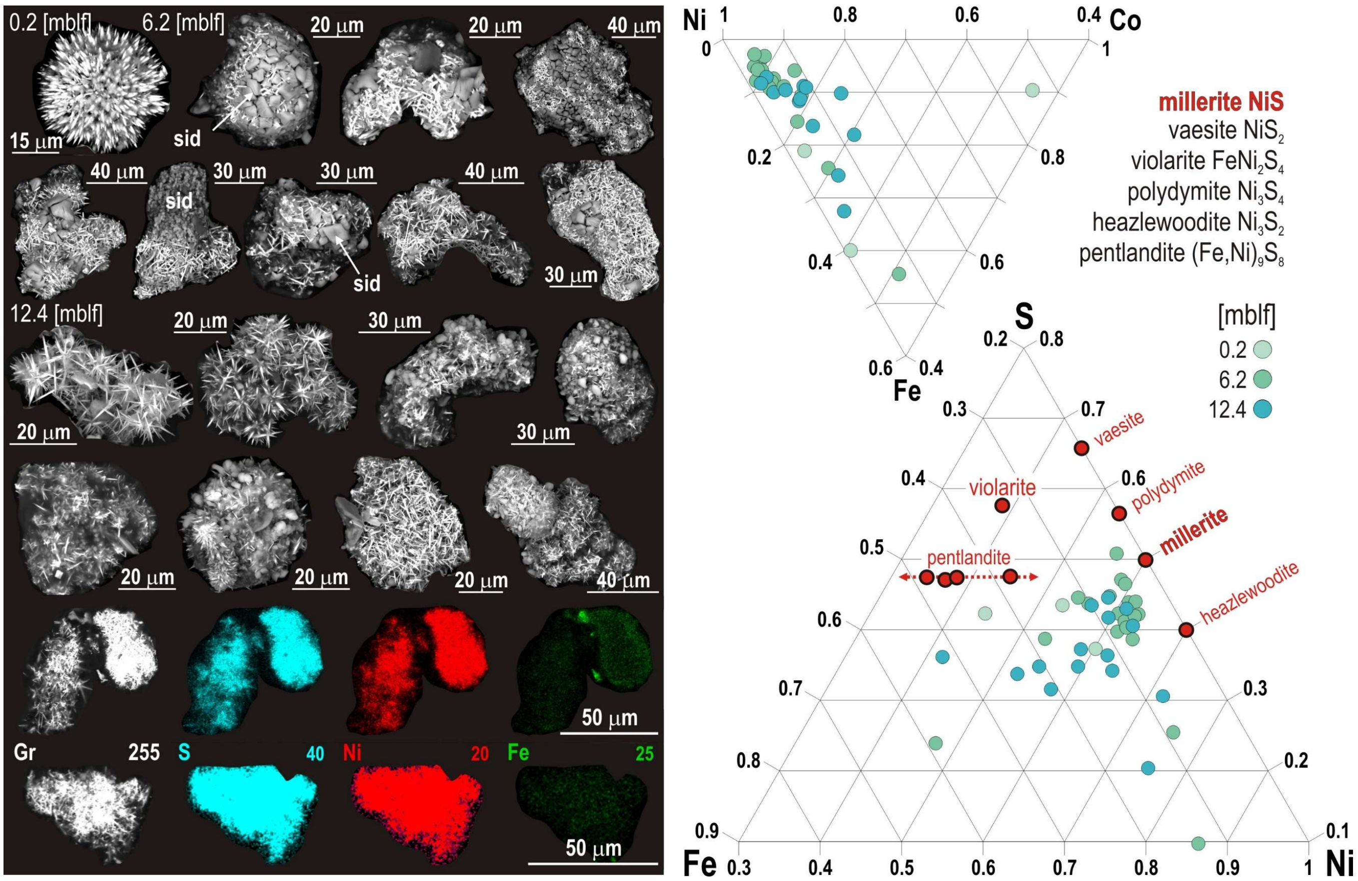

Supplementary Figure S1. Morphology and composition of authigenic millerites (NiS) extracted from Lake Towuti sediments. (A) Back-scattered electron images of acicular sulfide aggregates (upper rows) extracted from 0.2, 6.2, and 12.4 mblf, and EDX elemental mapping of millerite aggregates (bottom rows) depicting (from left to right): intensity (grey), and relative concentrations of sulfur (blue), nickel (red) and iron (green). Most aggregates display concentric growth fabrics composed of acicular crystals which are gradually overgrown by siderite (sid). Scale bars are 15–50 µm. **(B)** Ternary diagrams of EDX punctual analyses showing the relative atomic proportions of Ni-Co-Fe (top) and Ni-Fe-S (bottom). Red labels correspond to the stoichiometric compositions of some common nickel–iron sulfides. The figure is modified from Vuillemin et al., 2023.

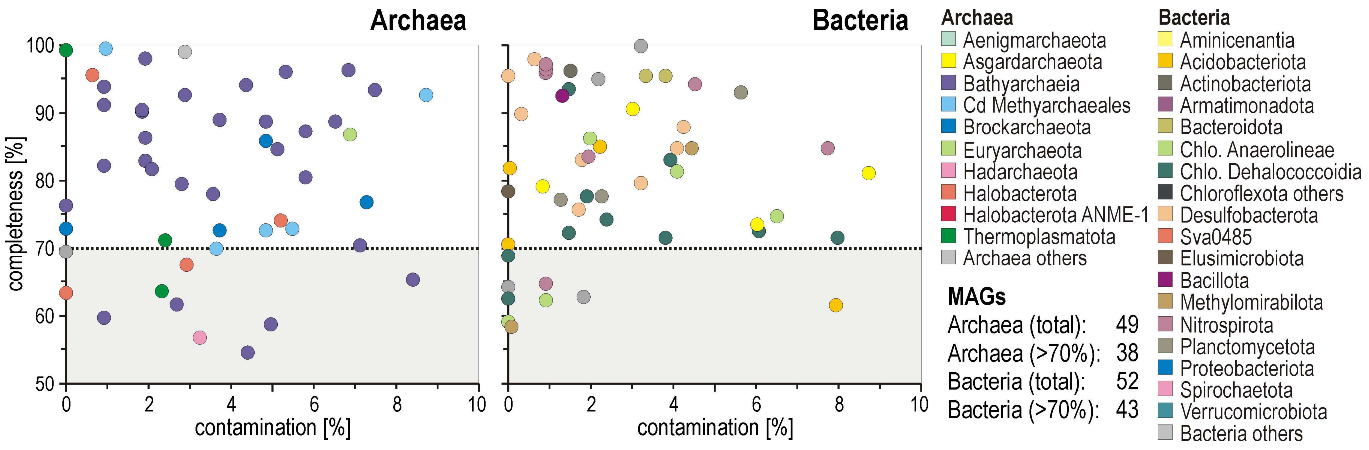

Supplementary Figure S2. Determination of metagenome-assembled genomes (MAGs) quality. Scatter plots of MAGs colored by phyla in terms of % contamination (x axis) versus % completeness (y axis); dashed lines mark the threshold for good-quality (>70 % completeness; <10 % contamination) MAGs. Only good-quality MAGs (i.e. 38 Archaea, 43 Bacteria) were selected for downstream analysis.

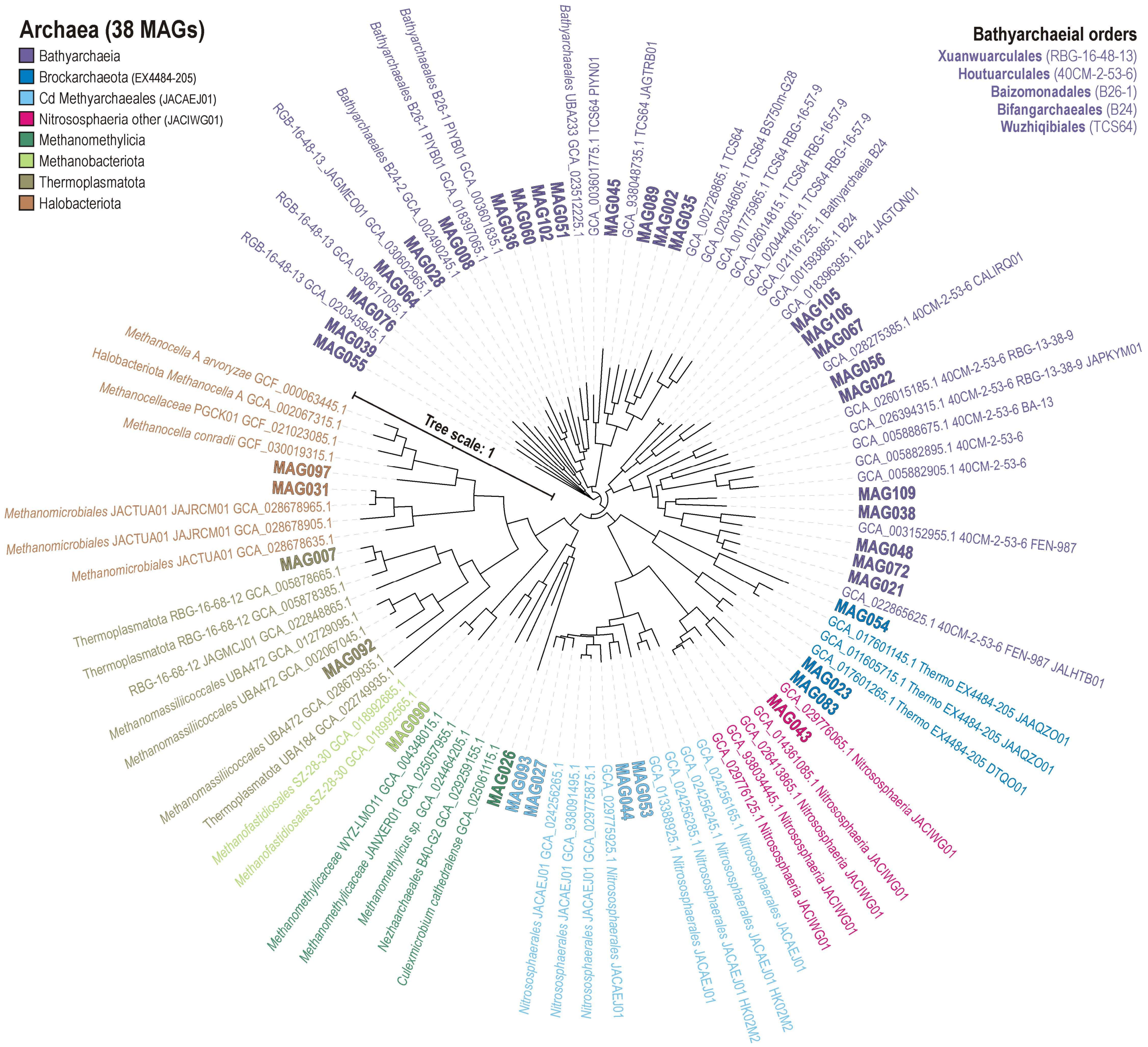

Supplementary Figure S3. Phylogenetic tree based on 16 concatenated ribosomal protein markers for archaeal MAGs. Phylogenetic tree based on 16 concatenated ribosomal protein sequences extracted from all 38 good-quality (>70 % completeness; <10 % contamination) metagenome-assembled genomes (MAGs) assigned to Archaea and 62 representative MAGs used as reference extracted from the GTDB database (Parks et al., 2022). Phylogenetic distances were computed using open scripts and published protocol (Graham and Tully, 2018; Graham et al., 2018). Tree visualization was achieved using iTOL v. 6 (Letunic and Bork, 2024).

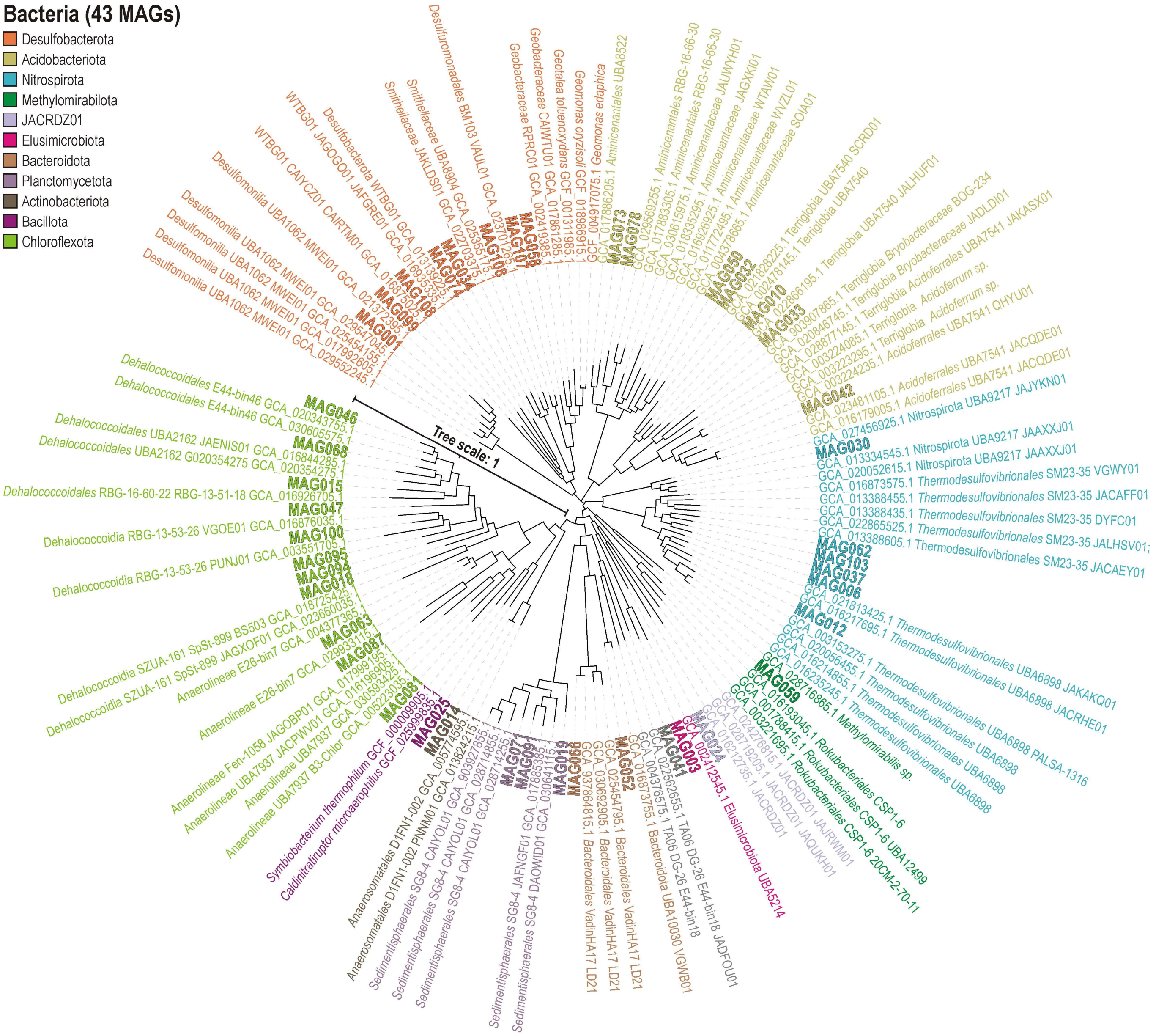

Supplementary Figure S4. Phylogenetic tree based on 16 concatenated ribosomal protein markers for bacterial MAGs. Phylogenetic tree based on 16 concatenated ribosomal protein sequences extracted from all 43 good-quality (>70 % completeness; <10 % contamination) metagenome-assembled genomes (MAGs) assigned to Bacteria and 87 representative MAGs used as reference extracted from the GTDB database (Parks et al., 2022). Phylogenetic distances were computed using open scripts and published protocol (Graham and Tully, 2018; Graham et al., 2018). Tree visualization was achieved using iTOL v. 6 (Letunic and Bork, 2024).

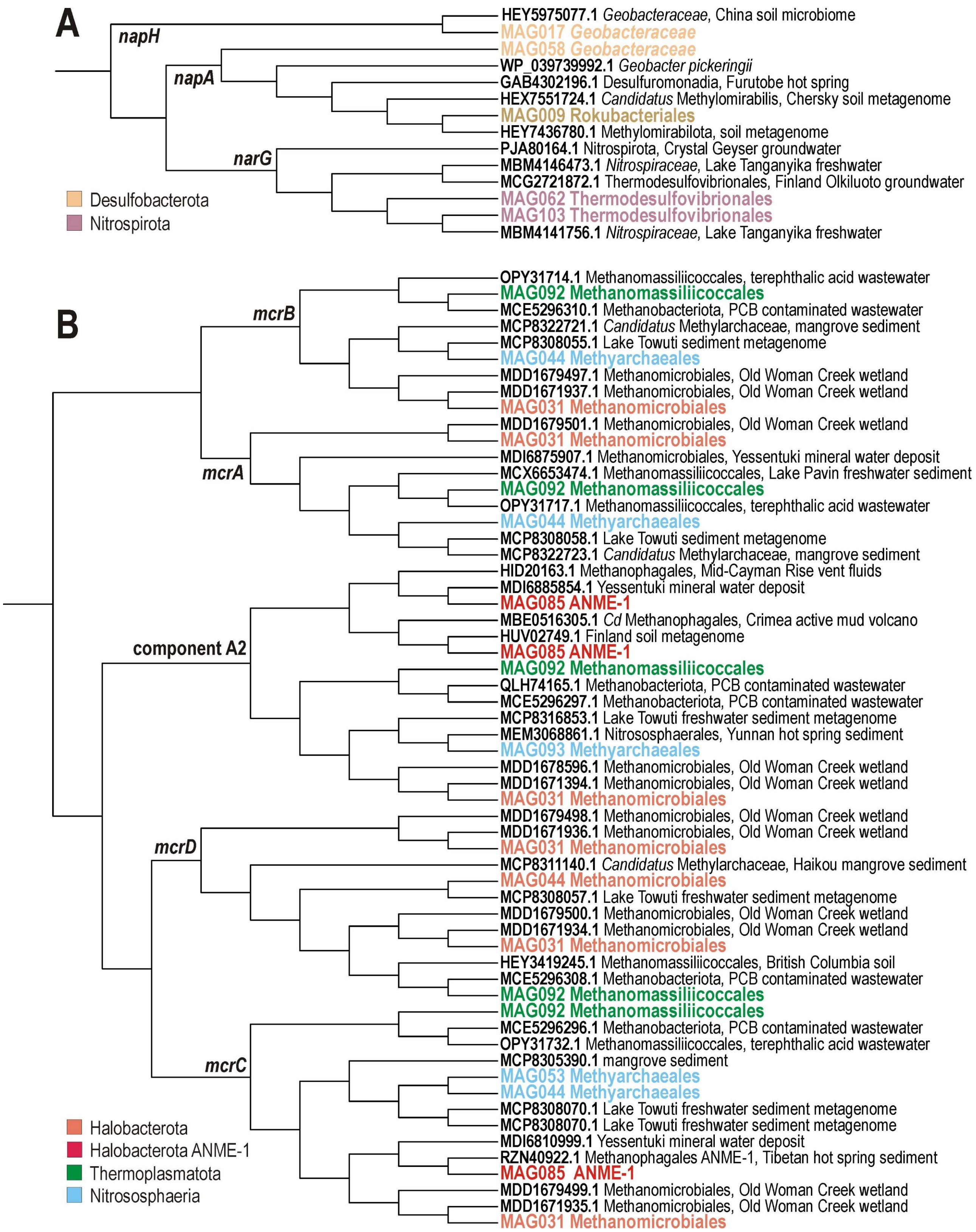

Supplementary Figure S5. Phylogenetic trees of respiratory and periplasmic nitrate reductase, and methyl-coenzyme M reductase protein-encoding genes. PhyML maximum likelihood tree of open reading frames encoding conserved regions extracted from metagenome-assembled genomes, with: (**A**) the respiratory (*narG*) and periplasmic nitrate reductase subunit alpha (*napA*) and iron-sulfur protein (*napH*); and (**B**) methyl-coenzyme M reductase subunit alpha (*mcrA*), beta (*mcrB*), gamma (*mcrC*), delta (*mcrD*) and component A2. The phylogenetic trees are based on 100 bootstrap replicates with BLOSUM62 as the evolutive model (Gouy et al., 2020; Guidon et al., 2010). Boldface type signifies accession numbers to the NCBI database.

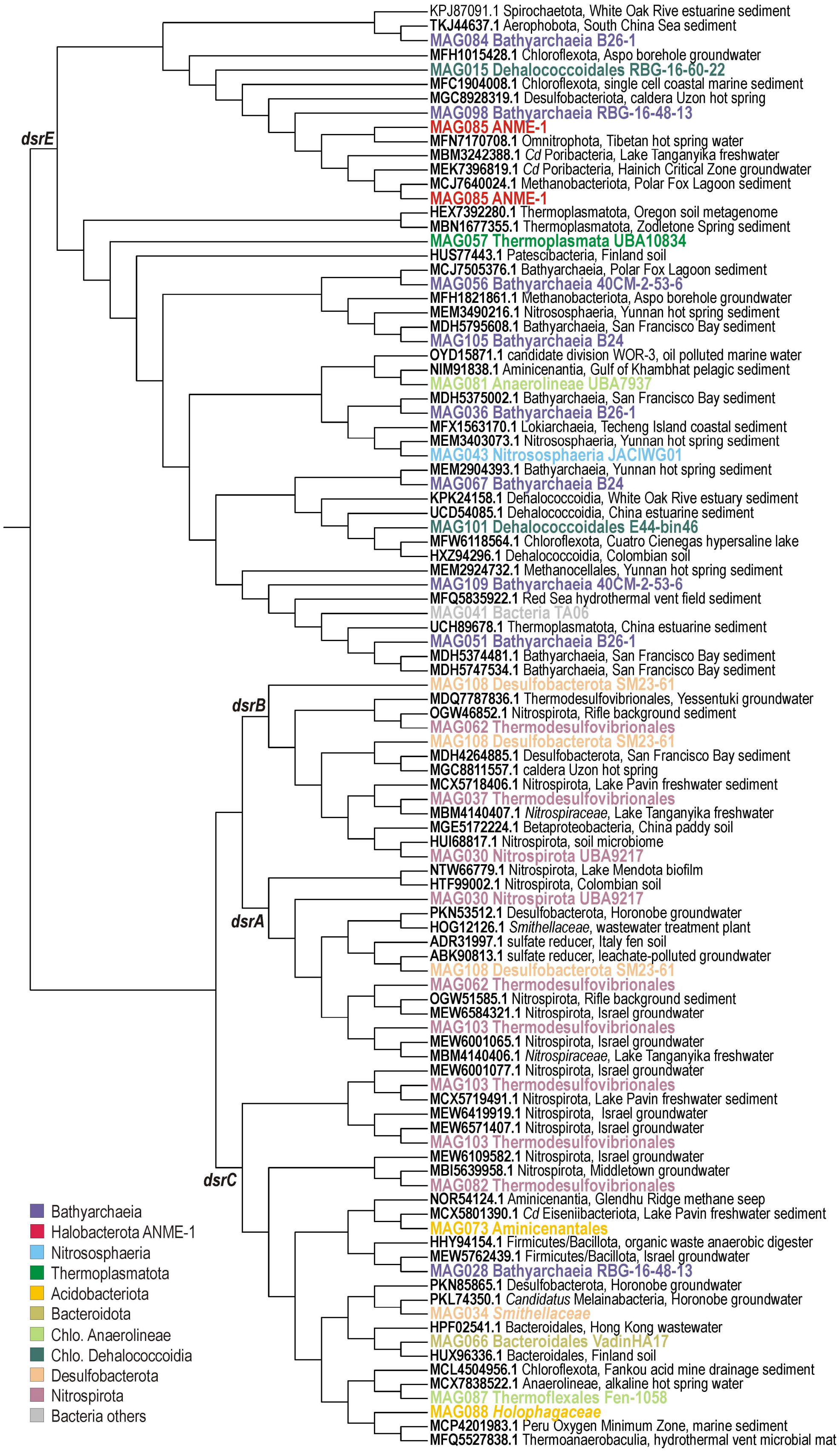

Supplementary Figure S6. Phylogenetic tree of dissimilatory sulfite reductase protein-encoding genes. PhyML maximum likelihood tree of open reading frames encoding conserved regions of the dissimilatory sulfite reductase subunit alpha (*dsrA*), beta (*dsrB*), gamma (*dsrC*), delta (*dsrD*) and epsilon (*dsrE*) extracted from metagenome-assembled genomes. The phylogenetic tree is based on 100 bootstrap replicates with BLOSUM62 as the evolutive model (Gouy et al., 2020; Guidon et al., 2010). Boldface type signifies accession numbers to the NCBI database.

Supplementary Table S1. List of enzymatic names and abbreviations for functional marker genes predicted from *de novo* assembly of metagenome-assembled genomes.

Processes	Enzymes	Gene abbreviations
Sulfur cycling	sulfate adenylyltransferase	*sat I-II*
	adenylylsulfate kinase	*APS-kn*
	adenylylsulfate reductase subunit alpha and beta	*aprAB*
	dissimilatory sulfite reductase subunit alpha and beta	*dsrAB*
	dissimilatory sulfite reductase subunit gamma	*dsrC*
	dissimilatory sulfite reductase subunit E	*dsrE*
	anaerobic sulfite reductase subunit alpha	*asrA*
	polysulfide reductase /thiosulfate reductase subunit alpha	*psrA/phsB*
	sulfhydrogenase (I and II)	*hyd I-II*
	sulfide dehydrogenase subunit alpha and beta	*sudAB*
	dimethyl sulfoxide reductase	*dsmo*
	sulfur oxygenase reductase	*SOR*
	sulfur oxidation proteins	*SOX*
	sulfite oxidase	*suox*
	taurine dioxygenase	*tauD*
	assimilatory sulfite reductase complex	*cysJ*
Nitrogen cycling	respiratory nitrate reductase	*nar*
	periplasmic nitrate reductase (ferredoxin-type)	*nap*
	respiratory nitrite reductase	*nir*
	anaerobic nitric oxide reductase (flavorubredoxin)	*nor*
	nitrous oxide reductase	*nos*
	ammonia-forming cytochrome c nitrite reductase	*nrf*
Iron cycling	outer membrane c-type cytochrome	*omc*
Methanogenesis	methyl coenzyme M reductase/coenzyme-B sulfoethylthiotransferase	*mcr*

Supplementary Table S2. Metadata on the *de novo* assembly of metagenomic reads into contigs for the 12 samples whose libraries were successfully sequenced. The 8 metagenomes obtained from the gravity core samples (Ruiz-Blas et al., 2024) were combined and processed as a single sample.

Sample ID	0-40 cmbsf (gravity core)	1.36 mbsf (S7959Nr1)	2.45 mbsf (S7959Nr200)	5.55 mbsf (S7959Nr500)	9.50 mbsf (S10644Nr2)
**Number of assembled reads**	203538874	15207684	9350366	5098948	42215204
**Assembled reads [%]**	33.70	27.08	21.76	47.26	45.65
**Number of scaffolds**	389130	27909	17219	1619	56988
**Number of contigs**	499817	42116	24266	2364	68157
**Contig length [bps]**	1149529556	77824769	49325330	9711366	179823849
**Contig N50**	87505	7518	4414	386	10354
**Contig L50**	2967	2312	2622	6583	3490
**Number of predicted genes**	1347949	98920	65044	10566	225843
**Number of predicted ORFs**	768007	49525	32927	6322	106726

Supplementary Table S3. Metadata on the *de novo* assembly of the 101 metagenome-assembled genomes (MAGs) obtained from the 12 metagenomic libraries successfully sequenced in this study, completed with two intracellular DNA samples (Vuillemin et al., 2018) sequenced at the Joint Genome Institute. In total, 81 good-quality (>70% completeness; <10% contamination) MAGs were selected for downstream analyses (i.e. 38 Archaea, 43 Bacteria).

MAG ID	MAG taxonomy (GTDB)	Completeness [%]	Contamination [%]	Genome size [bps]	Predicted ORFs [total number]
MAG001	Bacteria_Desulfobacterota_Desulfomonilia_UBA1062_UBA1062_MWEI01_	98.06	0.65	3534966	1771
MAG002	Archaea_Thermoproteota_Bathyarchaeia_TCS64_TCS64_	88.83	6.55	2896696	1924
MAG003	Bacteria_Elusimicrobiota_UBA5214_UBA5214_UBA5214_	78.59	0	1323698	655
MAG005	Archaea_Nanoarchaeota_Nanoarchaeia_Woesearchaeales_ARS49_	69.39	0	759848	347
MAG006	Bacteria_Nitrospirota_Thermodesulfovibrionia_Thermodesulfovibrionales_UBA6898_JACRHE01_	97.27	0.91	2845377	1819
MAG007	Archaea_Thermoplasmatota_Thermoplasmata_RBG-16-68-12_	71.09	2.4	1512469	776
MAG008	Archaea_Thermoproteota_Bathyarchaeia_B26-1_B26-1_PIYB01_	79.44	2.8	1196201	853
MAG009	Bacteria_Methylomirabilota_Methylomirabilia_Rokubacteriales_CSP1-6_	58.36	0.11	2518818	1911
MAG010	Bacteria_Acidobacteriota_Acidobacteriae_UBA7540_UBA7540_	85.2	2.22	3577421	1441
MAG011	Bacteria_UBA6262_UBA6262_WVXT01_WVXT01_	62.86	1.83	1021899	503
MAG012	Bacteria_Nitrospirota_Thermodesulfovibrionia_Thermodesulfovibrionales_UBA6898_	83.02	1.82	2387707	1809
MAG014	Bacteria_Actinobacteriota_Coriobacteriia_OPB41_D1FN1-002_D1FN1-002_D1FN1-002 sp005774595	96.25	1.53	2214235	894
MAG015	Bacteria_Chloroflexota_Dehalococcoidia_Dehalococcoidales_RBG-16-60-22_	83.22	3.96	1558178	1377
MAG017	Bacteria_Desulfobacterota_Desulfuromonadia_Geobacterales_Geobacteraceae_DSSF01_	84.73	4.09	3273745	2563
MAG018	Bacteria_Chloroflexota_Dehalococcoidia_E44-bin15_E44-bin15_Kmv38_	93.73	1.49	1919788	1442
MAG019	Bacteria_Planctomycetota_Phycisphaerae_Sedimentisphaerales_SG8-4_	77.32	1.27	4625528	3055
MAG021	Archaea_Thermoproteota_Bathyarchaeia_40CM-2-53-6_FEN-987_	84.58	5.14	1757357	1024
MAG022	Archaea_Thermoproteota_Bathyarchaeia_40CM-2-53-6_	87.38	5.83	1909306	1046
MAG023	Archaea_Thermoproteota_EX4484-205_EX4484-205_DTQO01_	85.93	4.85	2145706	887
MAG024	Bacteria_JACRDZ01	100	3.23	1791305	925
MAG025	Bacteria_Firmicutes_E_Symbiobacteriia	92.57	1.32	2767829	1446
MAG026	Archaea_Thermoproteota_Methanomethylicia_	72.66	3.74	1077727	436
MAG027	Archaea_Thermoproteota_Nitrososphaeria_Nitrososphaerales_JACAEJ01_JACAEJ01_	72.98	5.49	954058	678
MAG028	Archaea_Thermoproteota_Bathyarchaeia_RBG-16-48-13_	76.17	0	714390	500
MAG029	Archaea_Thermoproteota_Bathyarchaeia_B26-1_	59.59	0.93	1113089	897
MAG030	Bacteria_Nitrospirota_UBA9217_UBA9217_UBA9217_	96.59	0.91	3283158	1931
MAG031	Archaea_Halobacteriota_Methanomicrobia_Methanomicrobiales_JACTUA01_	95.62	0.65	1343643	1156
MAG032	Bacteria_Acidobacteriota_Aminicenantia_Aminicenantales_Aminicenantaceae_	90.8	3.04	3394519	2039
MAG033	Bacteria_Acidobacteriota_Acidobacteriae_Bryobacterales_Bryobacteraceae_	81.87	0.05	4984406	3052

MAG034	Bacteria_Desulfobacterota_Syntrophia_Syntrophales_Smithellaceae_Smithella_	87.9	4.25	2864430	2441
MAG035	Archaea_Thermoproteota_Bathyarchaeia_TCS64_TCS64_	90.19	1.87	2031107	1575
MAG036	Archaea_Thermoproteota_Bathyarchaeia_B26-1_BA1_	82.17	0.93	1354071	1041
MAG037	Bacteria_Nitrospirota_Thermodesulfovibrionia_Thermodesulfovibrionales_SM23-35_JACAEY01_	84.92	7.75	1789807	1746
MAG038	Archaea_Thermoproteota_Bathyarchaeia_40CM-2-53-6_	93.46	7.48	1705256	902
MAG039	Archaea_Thermoproteota_Bathyarchaeia_RBG-16-48-13_	82.77	1.94	1425730	702
MAG040	Bacteria_Chloroflexota_Dehalococcoidia_Dehalococcoidales_RBG-16-60-22_E44-bin89_	68.86	0	1088202	1088
MAG041	Bacteria_TA06_DG-26_E44-bin18_	95.1	2.2	2818740	1048
MAG042	Bacteria_Acidobacteriota_Acidobacteriae_Acidoferrales_UBA7541_	70.67	0	2134432	968
MAG043	Archaea_Thermoproteota_Nitrososphaeria_JACIWG01_JACIWG01_JACIWG01_	99.03	2.91	1983035	807
MAG044	Archaea_Thermoproteota_Nitrososphaeria_Nitrososphaerales_JACAEJ01_JACAEJ01_	99.51	0.97	1323719	772
MAG045	Archaea_Thermoproteota_Bathyarchaeia_TCS64_PIYN01_	88.83	4.85	2157504	1158
MAG046	Bacteria_Chloroflexota_Dehalococcoidia_Dehalococcoidales_E44-bin46_E44-bin46_	74.26	2.38	760154	720
MAG047	Bacteria_Chloroflexota_Dehalococcoidia_GIF9_	73.5	6.05	1726304	1613
MAG048	Archaea_Thermoproteota_Bathyarchaeia_40CM-2-53-6_FEN-987_	96.12	5.34	2718299	1503
MAG049	Archaea_Halobacteriota_Methanomicrobia_Methanomicrobiales_JACTUA01_	63.35	0	789364	670
MAG050	Bacteria_Acidobacteriota_Aminicenantia_Aminicenantales_Aminicenantaceae_	79.26	0.85	2560790	1704
MAG051	Archaea_Thermoproteota_Bathyarchaeia_B26-1_UBA233_	90.34	1.87	1778953	1328
MAG052	Bacteria_Bacteroidota_UBA10030_UBA10030_UBA10030_VGWB01_	95.61	3.84	4136366	1743
MAG053	Archaea_Thermoproteota_Nitrososphaeria_Nitrososphaerales_JACAEJ01_JACAEJ01_	72.68	4.85	1092369	741
MAG054	Archaea_Thermoproteota_EX4484-205_EX4484-205_JAAOZO01_	72.82	0	518791	184
MAG055	Archaea_Thermoproteota_Bathyarchaeia_RBG-16-48-13_JAGTRE01_	93.93	0.93	1418073	724
MAG056	Archaea_Thermoproteota_Bathyarchaeia_40CM-2-53-6_	86.41	1.94	1356328	744
MAG057	Archaea_Thermoplasmatota_Thermoplasmata_UBA10834_UBA10834_	63.65	2.34	1134443	1025
MAG058	Bacteria_Desulfobacterota_Desulfuromonadia_Geobacterales_Geobacteraceae_DSSF01_	89.91	0.32	2435330	1970
MAG059	Bacteria_Methylomirabilota_Methylomirabilia_DTKO01_DTKO01_	84.87	4.44	2228724	1388
MAG060	Archaea_Thermoproteota_Bathyarchaeia_B26-1_BA1_	96.26	6.85	1831757	1222
MAG061	Archaea_Thermoproteota_Bathyarchaeia_B26-1_BA1_	58.72	4.98	847882	737
MAG062	Bacteria_Nitrospirota_Thermodesulfovibrionia_Thermodesulfovibrionales_SM23-35_JACAEY01_	94.49	4.55	2334883	2144
MAG063	Bacteria_Chloroflexota_Anaerolineae_E26-bin7_E26-bin7_E26-bin7_	86.36	2	5350051	2674
MAG064	Archaea_Thermoproteota_Bathyarchaeia_RBG-16-48-13_	88.94	3.74	2155850	1085
MAG065	Bacteria_Zixibacteria_MSB-5A5_MSB-5A5_RBG-16-43-9_RBG-16-43-9_	64.22	0	1498892	1163
MAG066	Bacteria_Bacteroidota_Bacteroidia_Bacteroidales_VadinHA17_LD21_	95.48	3.33	4371783	2618
MAG067	Archaea_Thermoproteota_Bathyarchaeia_B24_JAGTQN01_	94.17	4.37	2985847	1251

MAG068	Bacteria_Chloroflexota_Dehalococcoidia_Dehalococcoidales_UBA2162_	71.62	7.98	1744094	1563
MAG069	Archaea_Thermoproteota_Bathyarchaeia_RBG-16-48-13_	54.62	4.42	1123522	638
MAG071	Bacteria_Planctomycetota_Phycisphaerae_Sedimentisphaerales_SG8-4_CAIYOL01_	77.84	2.27	1712550	1269
MAG072	Archaea_Thermoproteota_Bathyarchaeia_40CM-2-53-6_FEN-987_	80.34	5.83	1966623	1037
MAG073	Bacteria_Acidobacteriota_Aminicenantia_Aminicenantales_UBA8522_	81.23	8.74	3099362	2457
MAG074	Bacteria_Desulfobacterota_WTBG01_	83.64	1.94	2372928	1406
MAG076	Archaea_Thermoproteota_Bathyarchaeia_RBG-16-48-13_	78.07	3.58	1886766	958
MAG078	Bacteria_Acidobacteriota_Aminicenantia_Aminicenantales_RBG-16-66-30_	72.71	6.08	2607242	2045
MAG079	Archaea_Thermoproteota_Nitrososphaeria_A_Caldarchaeales_	69.83	3.64	1719991	820
MAG080	Bacteria_Chloroflexota_Anaerolineae_JAAYEA01_JAAYEA01_	62.39	0.92	3197773	1847
MAG081	Bacteria_Chloroflexota_Anaerolineae_UBA7937_	81.35	4.09	2054179	1258
MAG082	Bacteria_Nitrospirota_Thermodesulfovibrionia_Thermodesulfovibrionales_UBA6898_PALSA-1316_	64.88	0.91	1626987	1418
MAG083	Archaea_Thermoproteota_EX4484-205_EX4484-205_DTQO01_	76.86	7.28	2658692	1012
MAG084	Archaea_Thermoproteota_Bathyarchaeia_B26-1_B26-1_	61.6	2.7	860981	706
MAG085	Archaea_Halobacteriota_Syntropharchaeia_ANME-1_ANME-1_ANME-1-THS_	67.6	2.94	1018394	1005
MAG086	Archaea_Hadarchaeota_Hadarchaeia_	56.7	3.24	2068557	753
MAG087	Bacteria_Chloroflexota_Anaerolineae_Thermoflexales_Fen-1058_	74.7	6.52	5375065	3063
MAG088	Bacteria_Acidobacteriota_Holophagae_Holophagales_Holophagaceae_Holophaga_	61.71	7.95	2870976	2380
MAG089	Archaea_Thermoproteota_Bathyarchaeia_TCS64_TCS64_	70.31	7.12	2045644	1678
MAG090	Archaea_Methanobacteriota_B_Thermococci_Methanofastidiosales_SZ-28-30_	86.87	6.89	3289219	988
MAG091	Bacteria_Planctomycetota_Phycisphaerae_Sedimentisphaerales_SG8-4_JAFNGF01_	93.18	5.63	5917655	3352
MAG092	Archaea_Thermoplasmatota_Thermoplasmata_Methanomassiliicoccales_UBA472_	99.19	0	1957416	780
MAG093	Archaea_Thermoproteota_Nitrososphaeria_Nitrososphaerales_JACAEJ01_JACAEJ01_	92.72	8.74	1236851	759
MAG094	Bacteria_Chloroflexota_Dehalococcoidia_SZUA-161_	75.75	1.73	2500418	1849
MAG095	Bacteria_Chloroflexota_Dehalococcoidia_RBG-13-53-26_RBG-13-53-26_	71.55	3.83	1410813	1160
MAG096	Bacteria_Chloroflexota_Anaerolineae_E26-bin7_E26-bin7_E26-bin7_	59.21	0	2186870	1200
MAG097	Archaea_Halobacteriota_Methanocellia_Methanocellales_	74.02	5.23	1423933	907
MAG098	Archaea_Thermoproteota_Bathyarchaeia_RBG-16-48-13_	65.37	8.41	1450118	823
MAG099	Bacteria_Desulfobacterota_Desulfomonilia_UBA1062_UBA1062_MWEI01_	77.72	1.93	2806503	1674
MAG100	Bacteria_Chloroflexota_Dehalococcoidia_RBG-13-53-26_RBG-13-53-26_	72.44	1.49	1516189	1238
MAG101	Bacteria_Chloroflexota_Dehalococcoidia_Dehalococcoidales_E44-bin46_E44-bin46_	62.54	0	736580	672
MAG102	Archaea_Thermoproteota_Bathyarchaeia_B26-1_BA1_	81.72	2.1	1943224	1426
MAG103	Bacteria_Nitrospirota_Thermodesulfovibrionia_Thermodesulfovibrionales_SM23-35_JACAEY01_	96.82	0.91	1894507	1673
MAG105	Archaea_Thermoproteota_Bathyarchaeia_B24_JAGTQN01_	98.06	1.94	2136200	1074

MAG106	Archaea_Thermoproteota_Bathyarchaeia_B24_	92.72	2.91	2218003	1053
MAG107	Bacteria_Desulfobacterota_Desulfuromonadia_Desulfuromonadales_BM103_VAUL01_	95.48	0	2764156	1965
MAG108	Bacteria_Desulfobacterota_SM23-61_SM23-61_SM23-61_JACRCA01_	79.65	3.23	3590747	2729
MAG109	Archaea_Thermoproteota_Bathyarchaeia_40CM-2-53-6_	91.12	0.93	1336807	873

Supplementary References

Bushnell, B. (2014) BBMap: A fast, accurate, splice-aware aligner. https://sourceforge.net/projects/bbmap/

Cantalapiedra, C. P., Hernández-Plaza, A., Letunic, I., Bork, P., and Huerta-Cepas, J. (2021) EggNOG-mapper v2: Functional annotation, orthology assignments, and domain prediction at the metagenomic scale. *Mol. Biol. Evol.* 38, 5825–5829. doi: 10.1093/molbev/msab293

Gouy, M., Guindon, S., and Gascuel, O. (2010). SeaView version 4: A multiplatform graphical user interface for sequence alignment and phylogenetic tree building. *Mol. Biol. Evol.* 27, 221–224. doi: 10.1093/molbev/msp259

Graham, E. D., Heidelberg, J. F., and Tully, B. J. (2018). Potential for primary productivity in a globally-distributed bacterial phototroph. *ISME J.* 12, 1861–1866. doi: 10.1038/s41396-018-0091-3

Graham, E., and Tully, B. (2018). Building Phylogenetic Tree V.2. *Protocols.io* 9693, 1–10. doi: dx.doi.org/10.17504/protocols.io.q2pdydn (accessed April 22, 2024).

Guindon, S., Dufayard, J.-F., Lefort, V., Anisimova, M., Hordijk, W., and Gascuel, O. (2010). New algorithms and methods to estimate Maximum-Likelihood phylogenies: Assessing the performance of PhyML 3.0. *Syst. Biol.* 59, 307–321. doi: 10.1093/sysbio/syq010

Hyatt, D., Chen, G.-L., LoCascio, P. F., Land, M. L., Larimer, F. W., and Hauser, L. J. (2010) Prodigal: Prokaryotic gene recognition and translation initiation site identification. *BMC Bioinformatics* 11, 119. doi: 10.1186/1471-2105-11-119

Kang, D. D., Li, F., Kirton, E., Thomas, A., Egan, R., An, H., and Wang, Z. (2019) MetaBAT 2: An adaptive binning algorithm for robust and efficient genome reconstruction from metagenome assemblies. *PeerJ* 7, e7359. doi: 10.7717/peerj.7359

Letunic, I., and Bork, P. (2024). Interactive Tree of Life (iTOL) v6: recent updates to the phylogenetic tree display and annotation tool. *Nucleic Acids Res.* 52, W78-W82. doi: 10.1093/nar/gkae268

Nurk, S., Meleshko, D., Korobeynikov, A., and Pevzner, P A. (2017). MetaSPAdes: A new versatile metagenomic assembler. *Genome Res.* 27, 824–834. doi: 10.1101/gr.213959.116

Parks, D. H., Chuvochina, M., Rinke, C., Mussig, A. J., Chaumeil, P.-A., and Hugenholtz, P. (2022). GTDB: an ongoing census of bacterial and archaeal diversity through a phylogenetically consistent. rank normalized and complete genome-based taxonomy. *Nucleic Acids Res.* 50, D785–D794. doi: 10.1093/nar/gkab776

Ruiz-Blas, F., Bartholomäus, A., Yang, S., Wagner, D., Henny, C., Russell, J. M., et al. (2024). Metabolic features that select for Bathyarchaeia in modern ferruginous lacustrine subsurface sediments. *ISME Commun.* 4, ycae112. doi: 10.1093/ismeco/ycae112

Sieber, C. M. K., Probst, A. J., Sharrar, A., Thomas, B. C., Hess, M., Tringe, S. G., and Banfield, J. F. (2018) Recovery of genomes from metagenomes via a dereplication, aggregation and scoring strategy. *Nat. Microbiol.* 3, 836–843. doi: 10.1038/s41564-018-0171-1

Vuillemin, A., Horn, F., Friese. A., Winkel, M., Alawi, M., Wagner, D., et al. (2018). Metabolic potential of microbial communities from ferruginous sediments. *Environ. Microbiol.* 20, 4297–4313. doi: 10.1111/1462-2920.14343

Vuillemin, A., Morlock, M., Paskin, A., Benning, L. G., Henny, C., Kallmeyer, J., et al. (2023) Authigenic minerals reflect microbial control on pore waters in a ferruginous analogue. *Geochem. Perspect. Lett.* 28, 20-26. doi: 10.7185/geochemlet.2339

Wu, Y.-W., Simmons, B. A., and Singer, S. W. (2016) MaxBin 2.0: An automated binning algorithm to recover genomes from multiple metagenomic datasets. *Bioinformatics* 32, 605–607. doi: 10.1093/bioinformatics/btv638
